# Supplementary material for: Horizontal gene transfer and ecological interactions jointly control microbiome stability
Source: PLoS Biol. 2022 Nov 9;20(11):e3001847. doi: 10.1371/journal.pbio.3001847 (PMC9678337; doi:10.1371/journal.pbio.3001847)
Supplement: S1 Text — (DOCX) [file pbio.3001847.s001.docx]

**Horizontal gene transfer increases microbiome stability - Supplemental Information**

**Differing metrics of stability generally produce qualitatively similar results**

In our primary analysis we define microbiome stability based on the extent to which microbiomes decrease in abundance. However, other commonly used stability metrics include the change in community composition following a perturbation, or the time taken for a community to recover to its original state following a perturbation. We therefore assessed whether our key results held when quantifying alternative metrics of stability. Specifically, we calculated the Bray-Curtis dissimilarity between pre- and post-perturbation samples (capturing change in community composition) and the average time taken for each species to return to its approximate pre-perturbation value (mean $\left| X_{i}\left( t_{n} \right)-X_{i}\left( t_{pre} \right) \right|<0.01$).

Defining stability as the difference in community composition following a perturbation qualitatively recapitulated each of our key findings (Supplementary Fig A). Specifically, any resistance genes increased overall community stability, mobile resistance genes also increased background community stability, but immobile resistance genes decreased background community stability (note, change in community composition cannot be applied to the individual focal species). Similarly, defining stability as the time for species abundances to return to their pre-perturbation levels confirmed each of our key findings (Supplementary Fig B), but also suggested a. that in highly competitive communities immobile resistance genes also destabilize focal species (Supplementary Fig B.c) and b. prior selection can *decrease* the stability of background species in the presence of low-mobility resistance genes (Supplementary Fig B.e). Examining individual species trajectories revealed this first phenomenon was driven by large increases in the abundance of the focal species during the perturbation period, such that while the focal species did not suffer, it did shift from its pre-perturbation value. Meanwhile, the second phenomenon was driven by general slower growth, and thus slower return time of susceptible cells in the presence of low stressor levels.

**Qualitative results hold across varying community types**

To assess the generality of our theoretical results, we repeated each of our analyses within different regions of parameter space – systematically varying microbiome diversity (Supplementary Fig C) and interaction strengths (Supplementary Fig D), and allowing plasmids to be lost during segregation (Fig S5). Again we could recapitulate each of our major conclusions, namely, the broadly stabilizing effects of mobile resistance genes, and the potential for immobile resistance genes to destabilize certain community types. Notably, in contrast to our main results, in background communities with high diversity we found that immobile genes also destabilized highly cooperative communities (Fig 3G). This phenomenon is likely driven by the intrinsic high instability of high-diversity, highly cooperative communities.

**OTU IDs in soil microcosms**

| **OTU_ID** | **Species** |
| --- | --- |
| OTU_3 | *Pseudomonas sp.* |
| OTU_107 | *Pseudomonas fluorescens* |
| OTU_167 | *Pseudomonas stuzeri* |
| OTU_14 | *Pseudomonas umsongensis* |
| OTU_9 | *Bacillus megaterium* |
| OTU_19 | *Bacillus simplex* |
| OTU_174 | *Pseudomonas sp.* |
| OTU_131 | *Pseudomonas sp.* |
| OTU_103 | *Pseudomonas sp.* |
| OTU_148 | *Actinobacterium* |
| OTU_309 | *Pseudomonas sp.* |
| OTU_187 | *Pseudomonas sp.* |
| OTU_166 | *Methylobacterium* |
| OTU_239 | *Pseudomonas lini* |

**Table A. List of the 14 OTU’s identified in the positive control.** Putative taxonomic identification was performed using BLAST with the highest scoring hit for each OTU sequence listed here.

**Details of Bayesian linear models**

**Bayesian linear model 1** was fitted to explain the robustness of the total community in response to experimental treatments, as shown in Fig. 4C. Specifically the following effects were considered:

Resistance: the presence or absence of a resistance gene in that community; reference level: sensitive, alternative: resistant

Mobility: within communities containing a resistance gene, whether that gene was on the chromosome or a plasmid (either pQBR103 or pQBR57); reference level: chromosome, alternative: plasmid.

Exposure: whether or not the community had prior exposure to weak mercury selection; reference level: unexposed; alternative: exposed

All three were treated as population-level effects with Mobility nested within Resistance and Exposure in interaction with both. These effects were all fitted as Treatment contrasts. In addition, heteroscedasticity was accounted for by enabling residual standard deviation (sigma, on a log scale) to change with Exposure.

Broad priors were chosen for all effects. For population level effects, these were normal distributions centred on zero with standard deviation 0.1; and for the sigma effect (normal centred on 2, standard deviation 1), with respective intercepts normal with mean 0, standard deviation 1 and normal with mean -5, standard deviation 3. The degeneracy caused by the nesting of Mobility within Resistance (i.e. the fact that, in the absence of any resistance, Mobility is meaningless), was dealt with by fixing the priors for all meaningless parameters as a constant zero (e.g. the effect of having resistance on a plasmid in a susceptible population).

Population-level effects were fitted as shown in Table 3. In addition, the residual standard deviation was -5.4 [-5.7 – -5.1] (note the negative values are because these are on a log scale), on which the sigma effect of Exposure was found to be significantly positive (0.73 [0.30 – 1.16]).

**Table B** Population-level effects estimated by Bayesian linear model 1. Bolded estimates are significantly different from zero

| **Effect** | **Estimate (95% CI)** |
| --- | --- |
| Intercept | **-0.068** (-0.071 – -0.064) |
| Resistance | **0.030** (0.024 – 0.035) |
| Exposure | **0.022** (0.013 – 0.031) |
| Resistance:Mobility | **0.0065** (0.0018 – 0.011) |
| Resistance:Exposure | **-0.017** (-0.029 – -0.0043) |
| Resistance:Mobility:Exposure | -0.0035 (-0.014 – 0.0069) |

**Bayesian linear model 2** was the same as Bayesian linear model 1, except that separate stability values were taken for the focal and background communities, as shown in Fig. 4 D-E. There was therefore an additional effect:

Community: which community the value is for; reference level: background, alternative: focal.

This effect was treated as a population-level effect in interaction with all other population-level effects.

To account for the fact that focal and background community measurements could come from the same microcosm, this model used a group-level effect of microcosm (a different level for each individual microcosm) to fit the standard deviation among the 48 microcosms. Heteroscedasticity was then accounted for by allowing an uncorrelated difference in the standard deviation with Exposure; in addition, a sigma effect of Community was included.

The additional priors were also broad: for standard deviation effects this was a normal, centred on 0.1, with standard deviation 1. The prior for the Community sigma effect was normal centred on 0.1, with standard deviation 1.

Population-level effects were fitted as shown in Table 4. The group-level effect of microcosm was a standard deviation 0.0021 (0.000090 – 0.0051), which significantly increased with Exposure (0.011 [0.0072 – 0.016]). The residual standard deviation was -5.6 [-6.6 – -5.1]), which significantly increased in focal communities (1.33 [0.71 – 2.45]).

The value quoted in the main text for the effect of resistance on stability, given mobility is for the sum of the Resistance and Resistance:Mobility terms (calculated across draws from the posterior distribution)

**Table C** Population-level effects estimated by Bayesian linear model 2. Bolded estimates are significantly different from zero

| **Effect** | **Estimate (95% CI)** |
| --- | --- |
| Intercept | **-0.052** (-0.056 – -0.048) |
| Resistance | **-0.030** (-0.036 – -0.025) |
| Exposure | 0.006 (-0.004 – 0.017) |
| Community | **-0.17** (-0.18 – -0.16) |
| Resistance:Mobility | **0.046** (0.041 – 0.050) |
| Resistance:Exposure | **0.034** (0.019 – 0.048) |
| Resistance:Community | **0.23** (0.21 – 0.25) |
| Exposure:Community | **0.12** (0.10 – 0.14) |
| Resistance:Exposure:Mobility | **-0.031** (-0.044 – -0.018) |
| Reistance:Community:Mobility | **-0.050** (-0.064– -0.035) |
| Resistance:Exposure:Community | **-0.17** (-0.19 – -0.14) |
| Resistance:Exposure:Community:Mobility | 0.020 (-0.00015 – 0.040) |
|  |  |

**Bayesian linear model 3** was fitted to the proportion of the background community that was mercury resistant, before and after a mercury shock (Fig. 4B). These proportions were square-root arcsine transformed before analysis. Because each of these proportions was calculated from four counts of colony forming units (±gentamycin to distinguish foreground from background community and ±mercury to distinguish mercury sensitive from resistant), statistical noise meant that some values fell outside the range of 0-1. These values were truncated to their respective boundaries (0, or 1). The effect of this on the distribution meant that we fitted this model using robust regression (the ‘student’ family in the brms package), introducing a ν parameter for a *t* distribution of residuals.

The effects and priors in this model were the same as Bayesian linear model 2, except that, in place of Community (the data here is for the background community only) we tested a different effect:

Timing: when the measurements were taken relative to the mercury shock; reference level: before; alternative: after

Population-level effects were fitted as shown in Table 5. The group-level effect of microcosm was a standard deviation 0.063 (0.0022 – 0.18), which significantly increased with Exposure (0.11 [0.0045 – 0.31]). The residual standard deviation was -1.3 [-1.8 – -0.99]), which significantly increased after the mercury shock (0.56 [0.16 – 1.0]). The ν parameter was 17 (2.5 – 52).

**Table D** Population-level effects estimated by Bayesian linear model 3. Bolded estimates are significantly different from zero

| **Effect** | **Estimate (95% CI)** |
| --- | --- |
| Intercept | 0.027 (-0.11 – 0.18) |
| Resistance | 0.13 (-0.015 – 0.27) |
| Exposure | 0.027 (-0.12 – 0.17) |
| Timing | 0.13 (-0.015 – 0.28) |
| Resistance:Mobility | **0.21** (0.064 – 0.35) |
| Resistance:Exposure | 0.093 (-0.06 – 0.25) |
| Resistance:Timing | **0.22** (0.055 – 0.39) |
| Exposure:Timing | 0.00093 (-0.16 – 0.17) |
| Resistance:Exposure:Mobility | **0.21** (0.03 – 0.38) |
| Reistance:Timing:Mobility | **0.19** (0.017 – 0.36) |
| Resistance:Exposure:Timing | 0.05 (-0.13 – 0.22) |
| Resistance:Exposure:Timing:Mobility | -0.016 (-0.20 – 0.16) |

**Supplementary Figures**


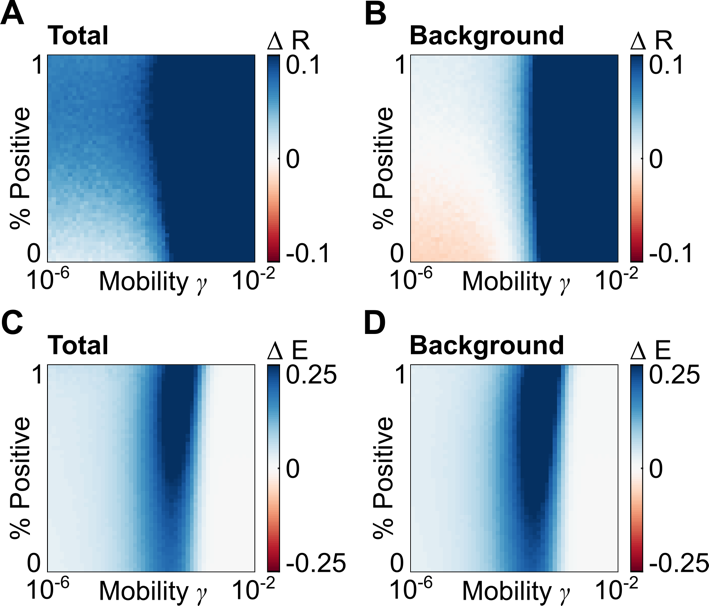


**Fig A. Defining stability based on community composition reproduces key results.** Recapitulating Fig. 3 analysis defining stability based on Bray Curtis dissimilarity. Throughout patch color represents mean ∆R or ∆E over 50 independent, 10-taxa communities, across a range of 51 % Positivity and $\gamma$ values. Other model parameters given in Table 1, underlying data at <https://github.com/katcoyte/hgt-microbiome-stability>.


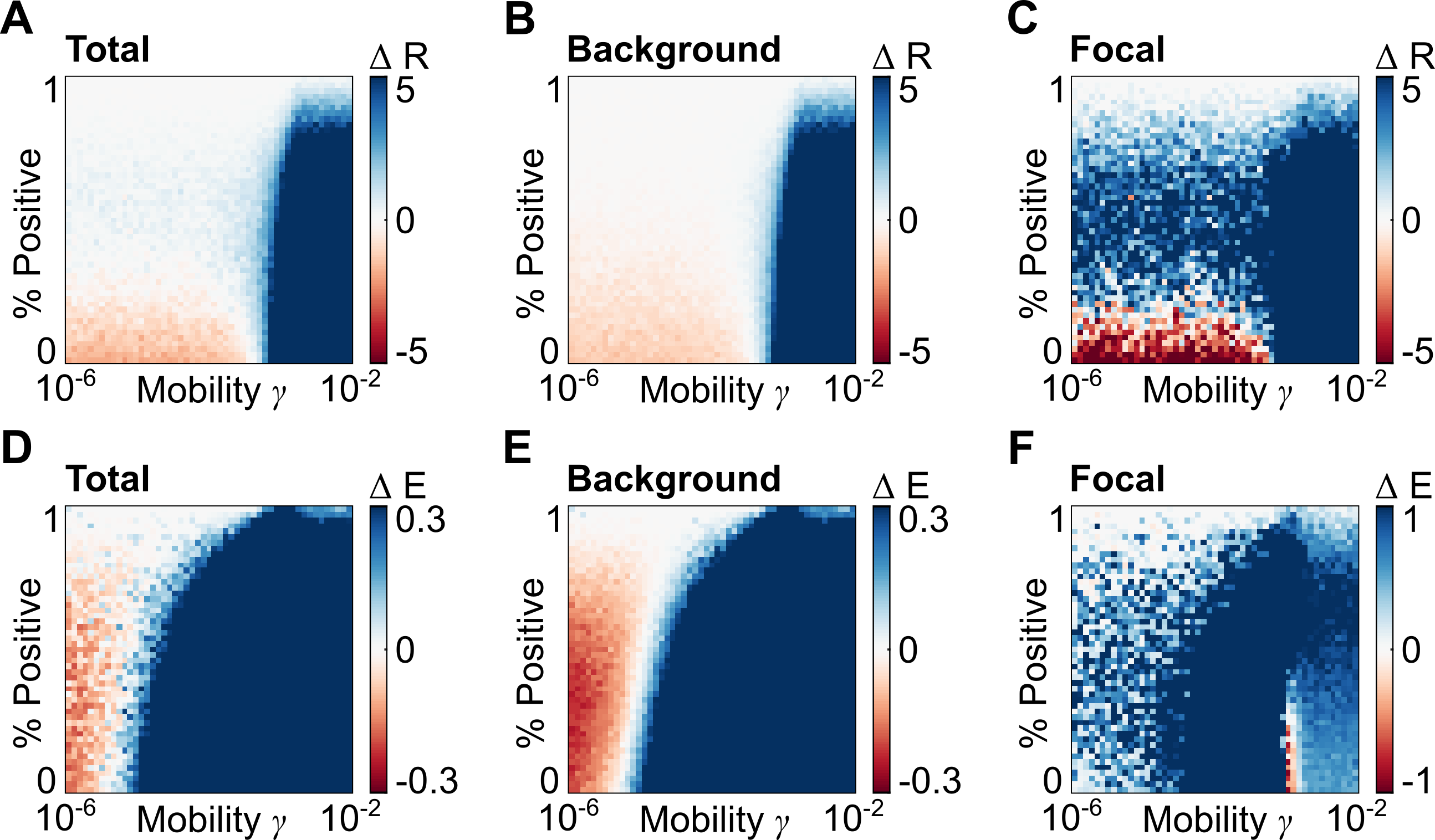


**Fig B Defining stability based on return time reproduces key results and reveals other trends.** Recapitulating Fig. 3 analysis defining stability based on average time for species abundances to return to their pre-perturbation levels. Throughout patch color represents mean ∆R or ∆E over 50 independent, 10-taxa communities, across a range of 51 % Positivity and $\gamma$ values. Other model parameters given in Table 1, underlying data at https://github.com/katcoyte/hgt-microbiome-stability.


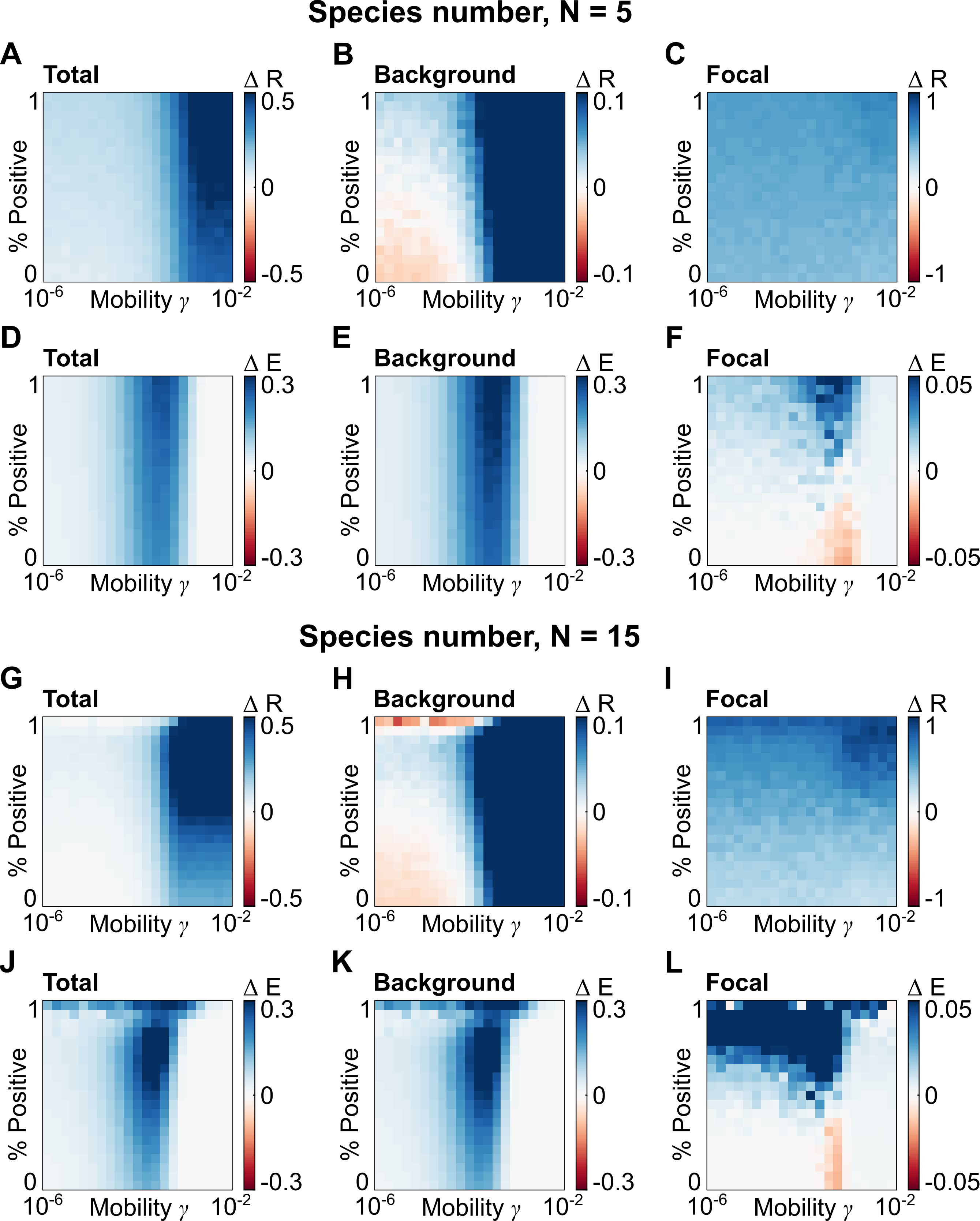


**Fig C. Varying microbiome diversity does not qualitatively change results. A-F.** Recapitulating Fig. 3 analysis for a five species community. **G-L.** Recapitulating Fig. 3 analysis for a fifteen species community. Our key results, such as the stabilizing effect of mobile resistance genes, and destabilizing effect of immobile resistance genes hold under differing community conditions. Notably, in high diversity communities immobile resistance genes also destabilize highly cooperative communities. This is because highly diverse, highly cooperative communities are particularly unstable, and are thus more vulnerable to changes in species abundances than less diverse or less cooperative communities (Coyte *et al* 2015). Throughout patch color represents mean ∆R or ∆E over 25 independent, 10-taxa communities, across a range of 21 Positivity and $\gamma$ values. Other model parameters given in Table 1, underlying data at https://github.com/katcoyte/hgt-microbiome-stability.


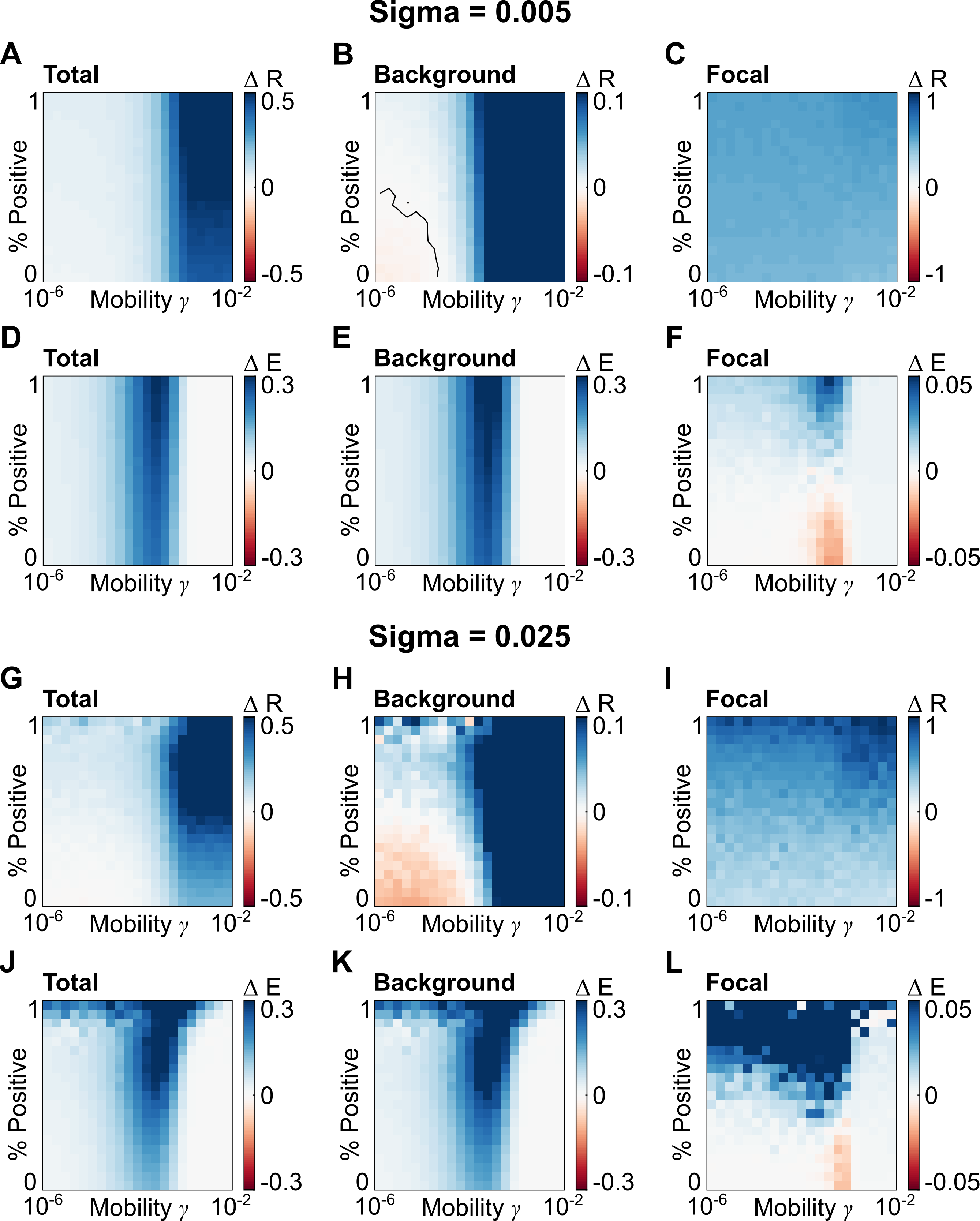


**Fig D. Varying interspecies interaction strength does not qualitatively change results. A-F.** Recapitulating Fig. 3 analysis for a community with weak interspecies interactions ($\sigma= 0.005$). **G-L.** Recapitulating Fig. 3 analysis for a community with strong interspecies interactions $(\sigma= 0.025$). Our key results, such as the stabilizing effect of mobile resistance genes, and destabilizing effect of immobile resistance genes hold under differing community conditions. Throughout patch color represents mean ∆R or ∆E over 25 independent, 10-taxa communities, across a range of 21 Positivity and $\gamma$ values. Other model parameters given in Table 1, to help with visualization the black line in B represents the 0 contour, underlying data at https://github.com/katcoyte/hgt-microbiome-stability.

**
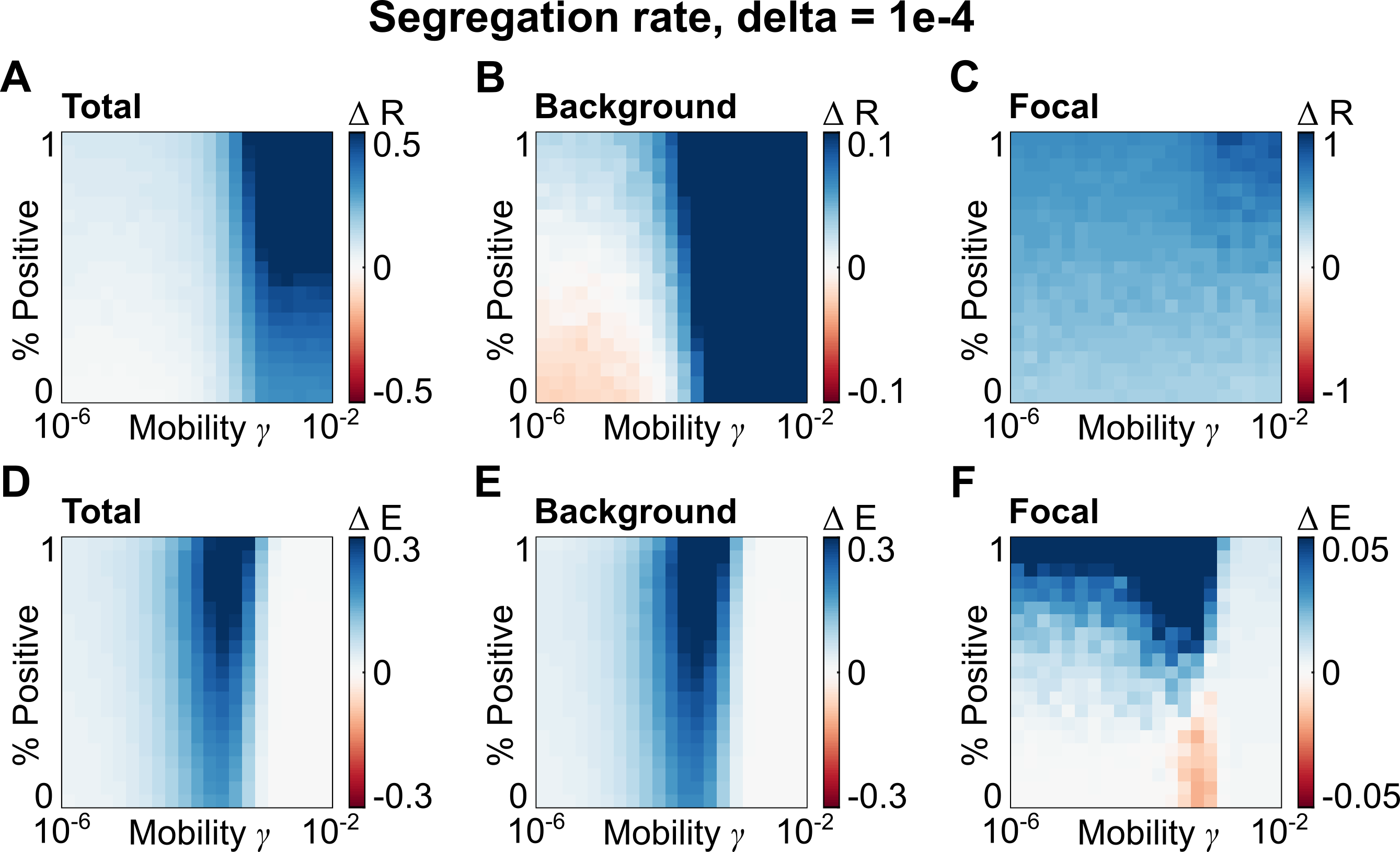
**

**Fig E. Allowing loss of resistance does not qualitatively change results. A-F.** Recapitulating Fig. 3 analysis when resistance genes can also be lost during segregation at a frequency$\delta= 1e^{-4}$. Throughout patch color represents mean ∆R or ∆E over 25 independent, 10-taxa communities, across a range of 21 Positivity and $\gamma$ values. Other model parameters given in Table 1, underlying data at <https://github.com/katcoyte/hgt-microbiome-stability>.

**
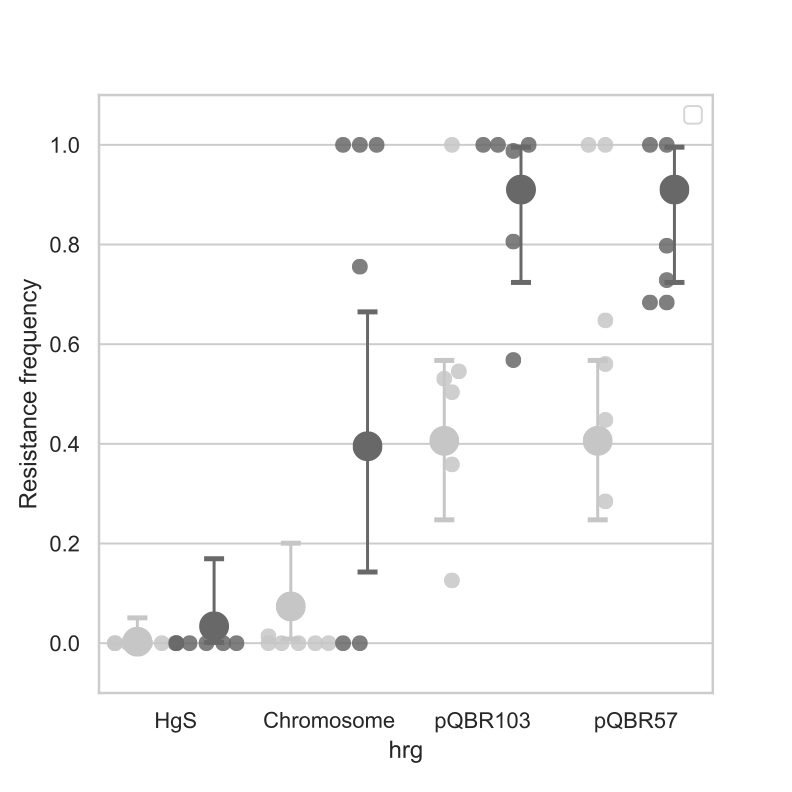
**

**Fig F. Resistance frequency in communities following prior mercury exposure.** Frequency of resistance within the background population before (light grey) and after (dark grey) the high-level mercury pulse following prior low-level mercury exposure. Calculated for each experimental condition (fully susceptible, HgS., and Chromosomal or plasmid-carried resistance, pQBR103, pQBR57). Underlying data at https://github.com/katcoyte/hgt-microbiome-stability

**
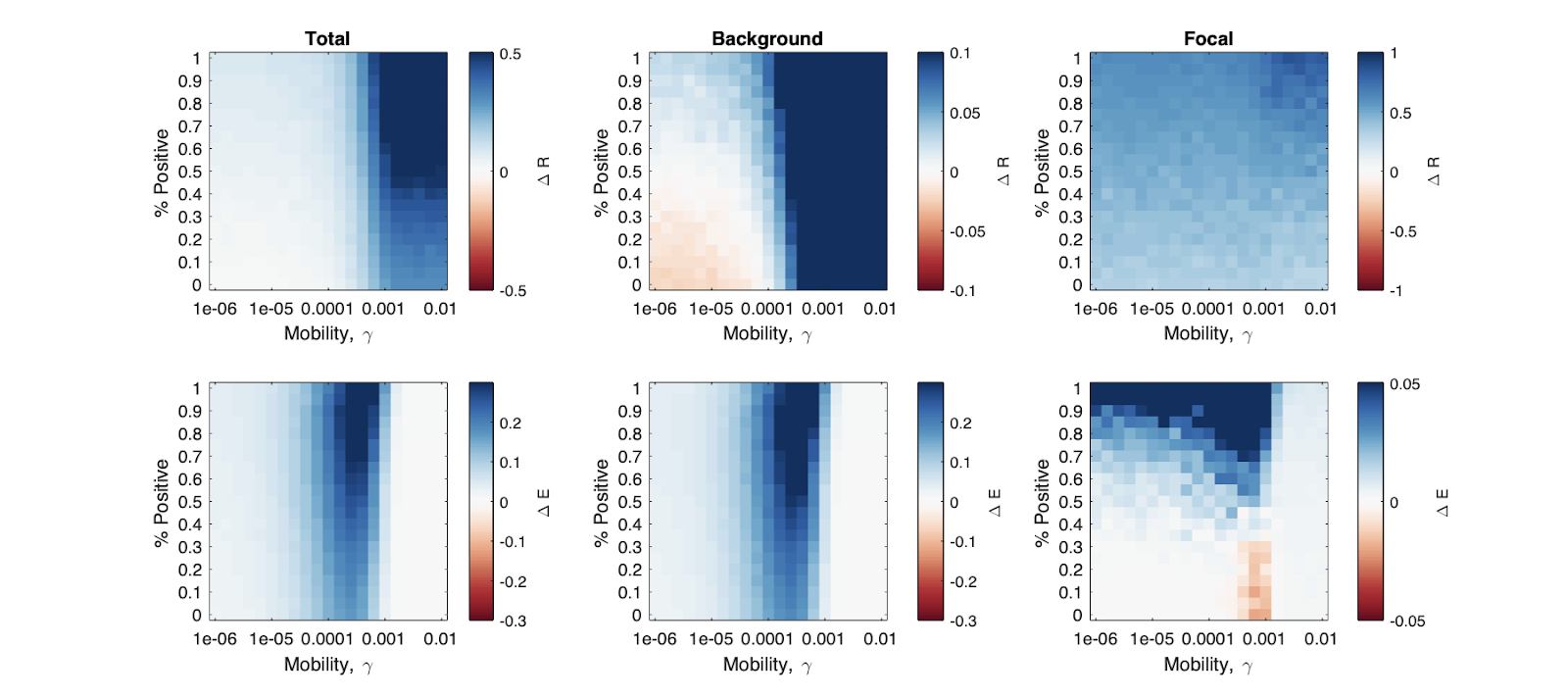
**

**Fig G. Modelling HGT only between interacting taxa.** Recapitulating Fig 3 analysis when resistance genes only transfer between taxa that are interacting ecologically (γ_ij_ >0 if |a_ij_| or |a_ji_| > 0). Patch color represents mean ∆R or ∆E over 25 independent, 10-taxa communities, across a range of 21 Positivity and $\gamma$ values. Other model parameters given in Table 1, underlying data at <https://github.com/katcoyte/hgt-microbiome-stability>.

**
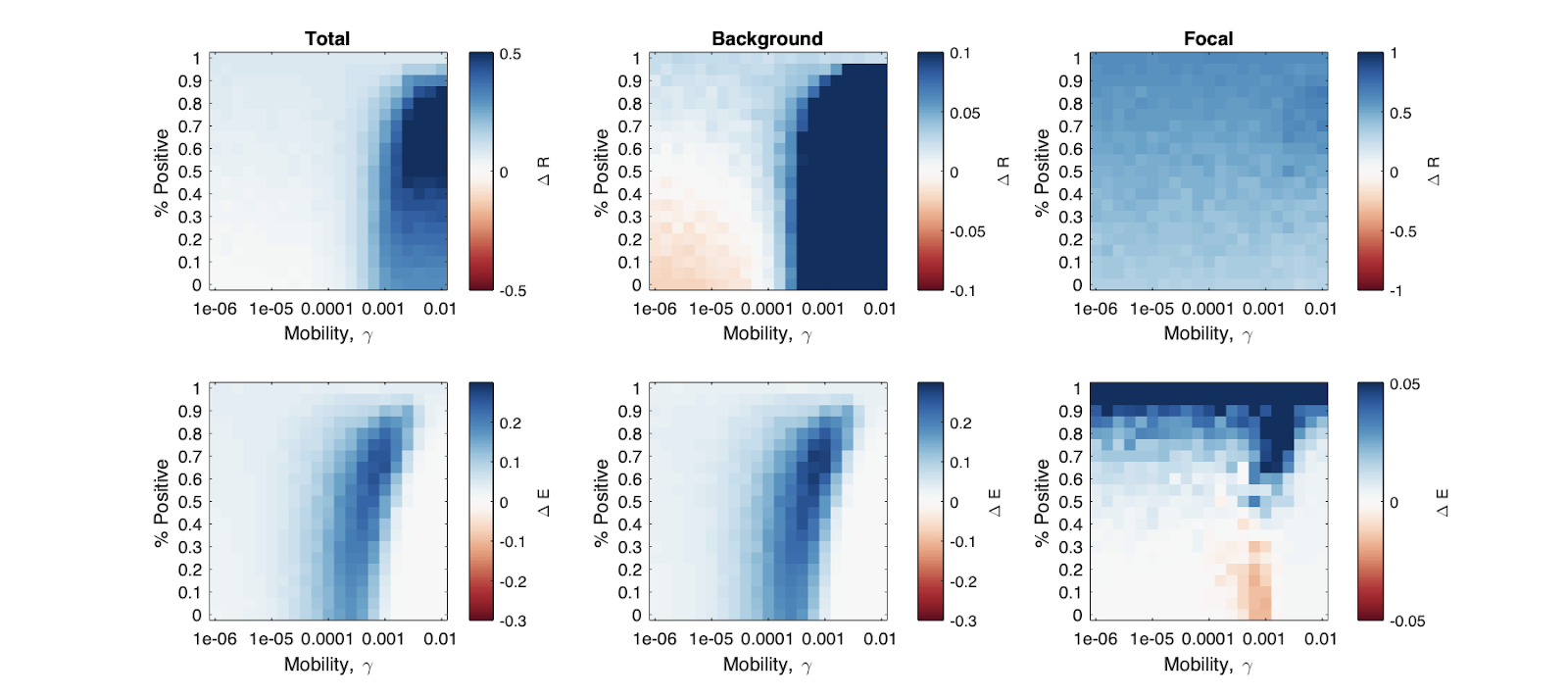
**

**Fig H. Modelling HGT only between negatively interacting taxa.** Recapitulating Fig 3 analysis when resistance genes only transfer between taxa that are interacting negatively (γ_ij_ >0 if a_ij_ or a_ji_ < 0). Patch color represents mean ∆R or ∆E over 25 independent, 10-taxa communities, across a range of 21 Positivity and $\gamma$ values. Other model parameters given in Table 1, underlying data at https://github.com/katcoyte/hgt-microbiome-stability.

**
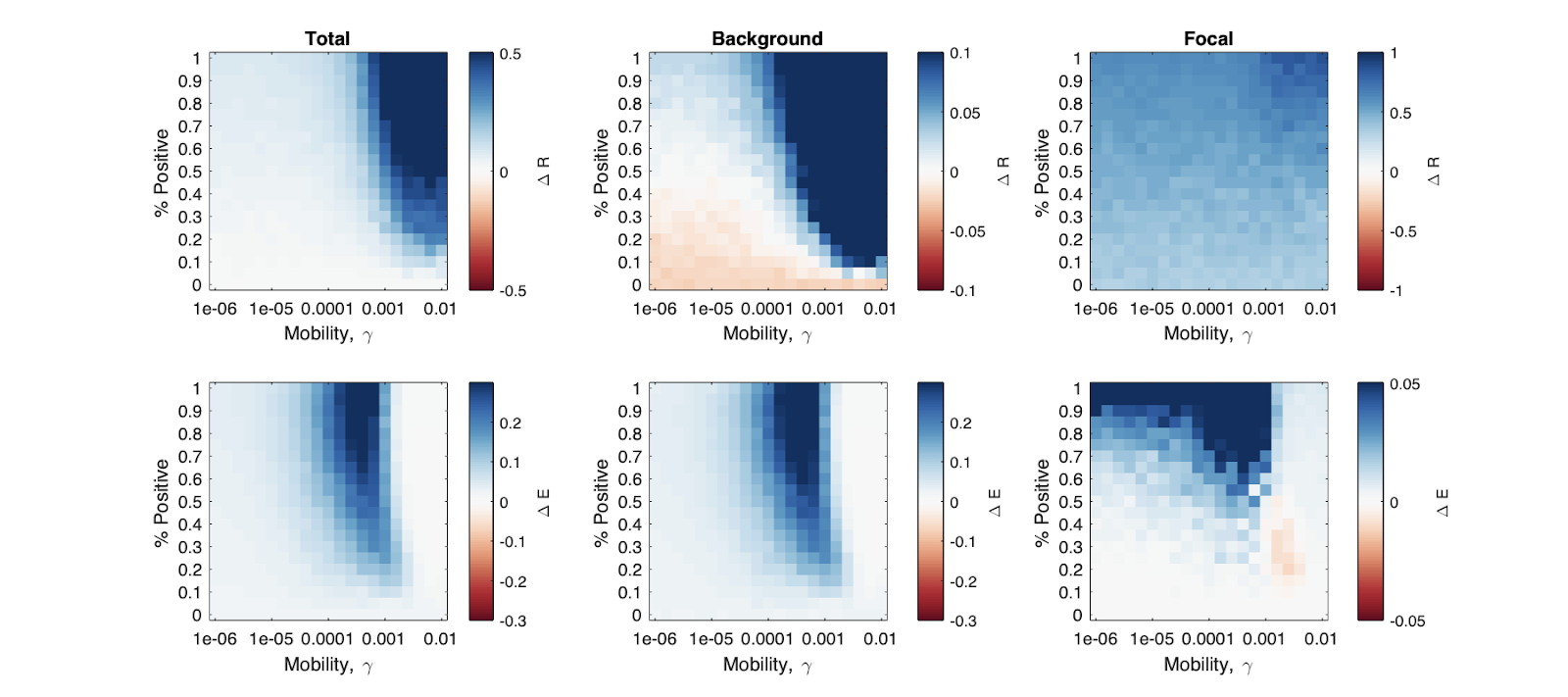
**

**Fig I. Modelling HGT only between positively interacting taxa.** Recapitulating Fig 3 analysis when resistance genes only transfer between species that are interacting positively (γ_ij_ >0 if a_ij_ or a_ji_ > 0). Patch color represents mean ∆R or ∆E over 25 independent, 10-taxa communities, across a range of 21 Positivity and $\gamma$ values. Other model parameters given in Table 1, underlying data at https://github.com/katcoyte/hgt-microbiome-stability.

**Fig J. Allowing smaller costs to immobile genes do not qualitatively change our results.** As immobile genes are often less costly than mobile ones, here we repeat the analysis in Fig 3, but allow a reduced cost for the least mobile resistance gene (setting c^immobile^ = c*0.1 for the case of $\gamma$ = 10^-6^). Throughout patch color represents mean ∆R or ∆E over 25 independent, 10-species communities, across a range of 21 Positivity and γ values. Other model parameters given in Table 1, underlying data at https://github.com/katcoyte/hgt-microbiome-stability.
