# Supplementary material for: Horizontal gene transfer and ecological interactions jointly control microbiome stability
Source: PLoS Biol. 2022 Nov 9;20(11):e3001847. doi: 10.1371/journal.pbio.3001847 (PMC9678337; doi:10.1371/journal.pbio.3001847)
Supplement: Attachment — Submitted filename: HGT_stability_reviewercomments_final.pdf [file pbio.3001847.s002.pdf]

We thank all three Reviewers for their helpful comments. We have addressed each of their concerns in our point-by-point below, and believe our manuscript is now far stronger following their feedback.

Reviewer #1: The topic is of general interest to a wide range of disciplines (microbiology, epidemiology, ecology). The combination of mathematical modelling with experiments advances our understanding of plasmid spread. The manuscript is, however, not clear enough in several ways which makes it particularly hard to assess whether the conclusions are correct.

We are glad the reviewer thinks our work has wide interest and advances our understanding. We have now substantially rewritten our manuscript to better explain both our methods and our conclusions.

The unclear part arises from the definition of stability and the way the authors deal with it. They define stability as the log of the ratio between species (or total) abundance before (Ab) and after (Aa)pertubation. It is , however, not clear when they measure Aa in their simulations. Is this at the end of the pertubation? That would be a wrong definition of stability. Stability is measured later on in the process, where it could have returned to (a new) stable state. In Figure 1 it seems that the Aa is measured at the wrong moment in time.

There are many different ways of measuring stability and these different measures tell us different things about community dynamics. In the main text, we focus on the difference in abundances immediately before and immediately after a perturbation. This is a very widely used and accepted definition of stability, often also referred to as Robustness, or Resistance (note, not to be confused with antibiotic resistance). In addition to this, in our Supplemental Information we also quantified a second metric of stability – the time taken for each species to return to their pre-perturbation value (SI Section 1, SI Fig 2). This metric captures behavior beyond the initial perturbation, including phenomena "later on in the process" such as communities returning to new stable states. Crucially, this second measure confirms each of our core conclusions, alongside revealing some more subtle additional dynamics. The similarities and differences between these two stability metrics are discussed in our new Supplemental Section *"Differing metrics of stability generally produce qualitatively similar results"*

We chose to focus on Robustness in our main text because it is the best match to our biological question (how does HGT affect how communities initially respond to a perturbation) and the most reliable to measure experimentally – allowing for proper comparison between theory and data. However, we understand that different readers may prioritize different metrics. As such, we have updated our text to make clearer our definition of stability, and bring more attention to the additional analyses in the SI:

> *"Although simple, these gLV models have been shown to well capture and predict the dynamics of both host-associated and environmental microbial communities[14,18–21].* ***However, previous work on the stability of these communities in the face of perturbations has assumed all taxa within any given microbiome are equally impacted by environmental perturbations such as antibiotics[13,14]. That is, microbiome stability has typically been assessed by examining how communities respond to uniform, instantaneous changes in each constituent taxa's abundance. As our goal is to explore dynamic variability in the impact of stressors on individual microbial taxa owing to resistance genes, we now*** *extended this basic model to explicitly incorporate a stressor that inhibits (or kills) susceptible cells and a potentially*

> *mobile resistance gene that protects cells encoding it, but at the cost of a reduced intrinsic growth rate.* ***This adjusted model allowed us to assess microbiome stability in the face of perturbations when the susceptibility of individuals to those perturbations varies between taxa and dynamically over time.***
>
> *…****More specifically, we focused primarily on one key measure of stability: the average decrease in taxa abundances following a perturbation, often termed community Robustness (Fig 1B). This measure allowed us to quantify the immediate response of the community to a perturbation. However, for completeness, we also quantified two further metrics of stability – the change in community composition induced by the perturbation (measured as Bray-Curtis dissimilarity), and the time taken for the community to return to its original state following the perturbation. Each of these alternative stability metrics produced qualitatively similar results (see Supplementary Information and Figures S1 and S2).”***

A second aspect that is not clear from the manuscript is what the authors mean exactly by stability after introduction of a plasmid. I do not think they mean introduction which would a pertubation by itself. They mean that they either search for a stable state with the plasmid presentand than pertubate it by an exposure to some external factor (mercury in the experiments). The same holds for the long term exposure which is not clearly defined.

We are not 100% clear on what specifically the reviewer finds unclear because they do seem to correctly understand the set up: we introduce a plasmid, allow the community to adjust to microcosm conditions, then perturb it with an external stressor (in our case mercury). In the case of pre-exposure we allow the community to equilibrate in the presence of a low stressor concentration, before exposing it to a high concentration pulse of the same stressor. In both cases we define stability as the response to this latter stressor perturbation.

Possibly the confusing factor here is the use of the term “introduce”, which implies the presence of the plasmid is itself an active perturbation that we are measuring. Therefore, we have modified the text throughout and instead refer to the effect of the “initial presence” of a resistance gene.

Alongside this, we have also updated our text to further clarify our simulation regime:

> *“More specifically, we could simulate the scenario in which a* ***given*** *microbiome* ***first has a fixed period of time to adjust to a new environment, and*** *is then briefly exposed to an external stressor such as a heavy metal or an antibiotic perturbation (Fig 1B).”*
>
> *“First, we simulated microbiome dynamics when all taxa were susceptible to the stressor, and then again when a randomly chosen focal taxon carried a gene* ***encoding resistance to the stressor. Depending upon its mobility, this resistance gene could spread into susceptible cells during both the initial adjustment period, and during the perturbation window.”***

In the results section the authors either make a mistake or I do not understand their idea of stability. They state that a negative ΔE or ΔR shows instability, but to my understanding of their definition all values that are not equal to 0 mean that the system is unstable. Therefore i do not

understand why they state that in figure 2D stability increases with mobility. I see an increasing instability because it is stable if Aa = Ab meaning the ratio = 1 thus log(1) = 0!

The reviewer has misunderstood these parameters. ΔE and ΔR do not reflect the raw stability values, but rather the change in stability from the initial presence of a resistance gene (ΔR), or following prior stressor exposure (ΔE). Raw stability values (as defined in Fig. 1B and measured in Fig. 4C-E) are indeed typically negative, indicating a reduction in abundance with perturbation, with zero indicating no such reduction (i.e. stability), However, negative values of ΔR or ΔE indicate that communities have got less stable, positive values indicate the community has got more stable (as in Fig. 1D and Fig 2D).

We have adjusted our text to make this clearer:

> *This process allows us to define two metrics: the change in microbiome stability resulting from the* ***initial presence*** *of a resistance gene, ΔR, and the change in microbiome stability resulting from prior exposure to low-level selection, ΔE (Fig 1D).* ***A positive ΔR indicates that a resistance gene increases stability, a negative ΔR indicates the resistance gene decreases stability (and equivalently for ΔE in terms of the effect of prior exposure upon stability).***

This really needs clarification and very thorough checking, because frankly as I got lost I stopped reading the paper much further.

We are sorry the reviewer did not read any further; we hope our clarifications will spur them to read on.

Some minor things that I found were:
Line 339: No explanation of choosing specific values for β

β_s and β_r represent the susceptibility of the sensitive and resistant strains respectively. As outlined in the methods, β_s=1 on the basis that all susceptible cells are entirely susceptible, β_r = 0.1 on the basis that even cells carrying a resistance plasmid will still be somewhat susceptible to antibiotics. We have edited the text to make this clearer:

> *More specifically, we set β_s =1 for the susceptible population, and β_r =0.1 for the resistant population,* ***on the basis that even cells harboring the resistance gene will still be slightly affected by the stressor***

Line 343/344: si is not specified. This is the self-interaction. Why is this the same for plasmid carrying and non carrying ? Do they ahve the same carrying capacity?

s_i =0.1, and is specified in Table 1. The cost of plasmid carriage is encoded in our model via a change to the intrinsic growth rate (-c), this translates to an adjustment in the intrinsic carrying capacity of -c/s.

Line 351: The model is not really new there are others that have defined a similar model
To our knowledge, while other models have described plasmid dynamics within a focal strain in the presence of an undefined "larger community" (e.g. Hall et al PNAS 2016) this is the first work to explicitly examine the spread and impact of plasmids within multispecies microbial

communities. However, if the reviewer is aware of any additional work we would welcome these references.

Line 359: rij should be ri
Thanks for catching this, we have adjusted accordingly.

Caption figure 2 I do not see error bars
Thanks for catching this, we have adjusted accordingly

Figure 3: different scales make the panel very hard to compare.
The different scales are necessary to visualize the full variability across parameter space between the different taxa (focal vs background).

Reviewer #2: This study addresses a very interesting question: How does horizontal transfer of resistance affect the stability of bacterial communities after a 'stressor' for which that gene confers resistance. The modeling is elegant and the experimental setup makes sense. The authors have a nice system to ask interesting questions. I have several major concerns however, as well as minor comments.

Thanks, we are happy the reviewer enjoyed the paper and, as outlined below, believe we can readily address each of their concerns

Major comments:

1. Overall, I have the impression that the authors - while having a very elegant and novel approach - make the outcomes of their simulations sound more unexpected and complex than they really are. In my opinion it is to be expected that if a resistance gene can transfer from a focal species to sensitive species, those species will do well after a pulse of the stressor for which the gene encodes resistance. Thus their abundance does not decrease as much as without having access to that resistance gene. It then follows that the community is more stable after a pulse of the stressor (with stability defined as a change in population density. It also is to be expected that this effect is greater in a cooperative than in a competitive community, where the donor of the gene shares its 'precious' resistance gene with competitors.

More often than not, scientific results are intuitive in hindsight. However, we cannot simply rely on intuition and opinion to ground our understanding. To our knowledge, none of our hypotheses have been formalized theoretically or tested either experimentally or in silico.

More importantly however, we disagree with the reviewer that all of our results are to be expected. Both the strong destabilizing effect of low-mobility resistance genes on background community members and the disadvantage to the focal species of carrying mobile genes may well be intuitive in hindsight, but they were certainly not a part of our initial hypotheses. Moreover, a key message of our study, that bulk community measures such as overall community abundance provide a poor understanding of the mechanisms driving microbiome stability, stands in stark contrast to the overwhelming majority of microbiome data analyses (although, as discussed in more detail below, we evidently did not make this message clearly enough in our original manuscript so have updated our text accordingly – see below for details of textual changes).

2. L. 195: "Experimental microbial communities reproduce theoretical predictions": This heading and other conclusions in the text seem to exaggerate the similarity between experimental results and simulation results: Some of the experimental results support the model, but not all. For example Fig. 4A doesn't distinguish between the resistance frequencies for communities with and without prior exposure.

Our experimental work reproduced all of our major theoretical predictions, including the positive effect of mobile resistance genes on microbiome stability, the surprising negative impact of immobile resistance genes, and the negative impact of HGT on original host cells. However, to avoid any overstatement, we have adjusted this heading to read:

“Experimental microbial communities reproduce **key** theoretical predictions”

Regarding Fig 4A, we assume the reviewer is referring to 4B because 4A is a schematic. 4B outlines the resistance frequencies in the absence of prior exposure. We have edited the legend of Fig 4 to make this clear and have now added a new supplemental figure, Fig S6, plotting the resistance frequencies in the presence of prior exposure (see below). Our apologies, we had intended to include this already.

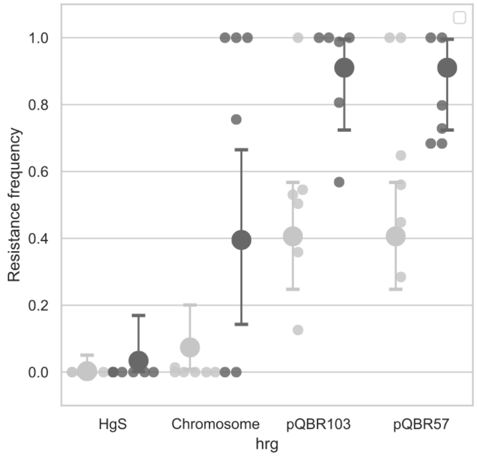

Fig S6. Resistance frequency in communities following prior mercury exposure. Frequency of resistance within the background population before (light grey) and after (dark grey) the high-level mercury pulse following prior low-level mercury exposure. Calculated for each experimental condition (fully susceptible, HgS., and Chromosomal or plasmid-carried resistance, pQBR103, pQBR57)

What was the effect of exposure on resistance (gen) frequency before the pulse? The model assumed 100%!

As shown by our new Fig S6 and original Figs 2C and 4B, in both our experiments and our models prior exposure increases resistance frequency, but overall resistance remains low, and resistance only reaches 100% in the model in the case of “super-mobile” plasmids.

Crucially, our model does not assume resistance frequency is 100% and we have adjusted our text to make this clearer:

> *For example, prior exposure to low-level stressor selection introduced a weak benefit to harboring the resistance gene prior to the perturbation,* ***enabling mobile resistance genes to spread and reach low but non-zero frequencies in*** *background taxa even at lower rates of gene mobility (Fig 2C)*

The authors did not see an effect of exposure on stability as expected from the model: L232, "a positive impact of gene mobility on overall microbiome stability, however, this effect was small (...) and did not change significantly with prior exposure (...)."

As outlined in the end of this sentence, the lack of impact of prior exposure on overall microbiome stability reflects the fact that total community stability is dominated by the behavior of the already resistant focal species. This is in line with our theoretical predictions. We have edited our text to make this point clearer:

> *"…and did not change significantly with prior exposure (estimated interaction effect between exposure and mobility, given resistance = -0.0035 [-0.014 – 0.0075 95% CI], BLM1).* ***Together these results confirmed key theoretical predictions that resistance genes increase stability at the scale of entire communities, but such coarse-scale measures are likely to ignore important differences between individual taxa and compartments of the microbiome****."*

3. Throughout the paper, for example L. 121: Overall microbiome stability does not seem a useful or correct measure here. It is defined as the difference in total number of cells before and after the perturbation: in this case with a chromosomal resistance (R), only the focal strain with the R gene survives, of course, and keeps the counts high. I would not call that overall stability of the community. When you look at individual species you see that the other two crashed, and thus their Delta R is zero, as expected. The authors say this much themselves at the end of the Discussion ~ L. 303: "Relying solely on coarse, whole-community metrics such as overall community abundances or diversity may mask striking differences between individual community members, and risks obscuring important eco-evolutionary dynamics." Here they did not even look at diversity but only at abundance.

We are confused by this comment because throughout the paper we highlight the shortcomings of using coarse overall community measures when species vary in their response to perturbation. Indeed, in their comment the reviewer has restated exactly one of the main messages that we wished to make! Namely, that looking at coarse metrics that average across an entire community will mask the dynamics of individual species. It is for precisely this reason that we split our analysis into focal (originally resistant) and background (originally susceptible) taxa, and illustrate that these follow radically different dynamics.

This is one of the central messages of our manuscript, and so we have made numerous adjustments to our abstract and main text make this point more strongly and clearly. E.g.:

> *“However, the impact of resistance genes upon the stability of individual taxa varies* ***dramatically*** *depending upon* ***the identity of individual taxa****, the mobility of the resistance gene and the network of ecological interactions within the community.”*

> ***“Remarkably however, w****hile mobile resistance genes* ***increased*** *overall microbiome stability, examining focal and background taxa separately revealed* ***radically*** *different* ***impacts of the resistance gene on individual taxa. That is,*** *background and focal species* ***showed*** *markedly different responses to resistance genes depending upon the precise manner in which taxa were interacting and the mobility of the resistance gene….*

> ***…This stark difference in dynamics between the community as a whole and that of background taxa reveals the critical importance of looking beyond average community properties when studying microbiome stability.”***

Related to this, we focus on abundance rather than diversity as this enables us to look at the focal taxon separately to the background taxa (we can’t calculate the diversity of a single taxon). However, we did include an in silico analysis of community dissimilarity (Bray-Curtis dissimilarity) in our original SI and have now edited our text to bring this more clearly to the reader’s attention:

> ***“However, for completeness, we also quantified two further metrics of stability – the change in community composition induced by the perturbation (measured as Bray-Curtis dissimilarity), and the time taken for the community to return to its original state following the perturbation. Each of these alternative stability metrics produced qualitatively similar results”***

4. L281: Again, I don't think one can say that a chromosomal resistance gene increases community stability. Thus instead of 'particularly when encoded on highly mobile genetic elements ', I think community stability should be redefined (see above) and it should read 'only when'.

As discussed above, this is precisely the point we wanted to make - chromosomal resistance *does* increase stability when averaging across the entire community (Figs. 3B, 4C, as is typically done in most microbiome studies) - however, in competitive communities it decreases the stability of initially susceptible background taxa (Figs 3C, 4D). See above for examples of adjustments to our text to make this point more clearly.

5. Another major concern is about the range of values for the conjugation rate gamma: 10^-6 to 10^-2 does not seem realistic to me. In my experience some of the most efficiently transferring plasmids have values lower than the lowest value used here, 10^-6. While it makes sense that increasing transfer rates will increasingly provide resistance to the background community and thus improve stability, the question how rapidly this can happen at known conjugation rates in the face of plasmid cost (see also next comment)

To address the reviewer’s fundamental scientific concern: As demonstrated by Fig 4B, we find that resistance transfers rapidly between species within real communities, despite the cost of harboring a plasmid.

To our particular parameter choice within the model: in line with previous theoretical work (e.g. Allesina and Tang, Nature 2012, Coyte et al Science 2015), we use a standard set of non-dimensional ecological parameters, setting the growth rate and interaction parameters such that

at equilibrium in the absence of any stressor each species reaches a density of ~1. We then choose our evolutionary parameters to scale appropriately with these.

Crucially, in this system, the key scale is the overall probability that a given cell will receive a plasmid at any given time. This is set not just by the baseline conjugation rate, but also by the number of cells harboring a plasmid at any given time - that is, gamma x X*

In previous work with our experimental system (Hall et al PNAS 2016) we found equilibrium densities X* ~= 10^8 and between-species conjugation rates of approximately gamma = 10^-13 /day, giving an overall probability of transfer of around 10^-5. Thus, to achieve an equivalent probability rate for an equilibrium population of size X* = 1, we set our gamma around this range.

We prefer to use these standard ecological parameters to keep our results comparable with previous work on microbiome stability, and to avoid anchoring ourselves within any particular system, though we note that there is great diversity in the literature in reporting conjugation rates, their dimensions and their absolute values (e.g. see Huisman et al. 2022). We have therefore added further text in our material and methods to explain this choice of parameter range:

> ***"To allow comparison to previous work, throughout we choose a standard set of ecological parameters for our model, then select our evolutionary parameters to scale accordingly. For example, we vary our rate of plasmid transfer gamma across [10^-6, 10^-2], covering scenarios from effectively immobile genes, through those with an equivalent probability of inter-taxa plasmid acquisition to that observed in Reference 24 [Hall et al 2016], to rare "super-mobile" plasmids. Importantly, however, we find qualitatively equivalent results when varying each of these key parameters (Figs S3-5, 10)."***

6. What were the assumptions of the cost of the increasingly mobile resistance (R) genes versus the chromosomal gene? It seems that only one cost value was used for the R gene. However, one would typically assign a lower fitness to the strain with plasmid-encoded R gene than the one with chromosomal R gene. Cost should depend on the gene being mobile or not mobile, since plasmids have a cost on top of the gene. Again, this would require an even higher transfer rate to compensate for the higher cost of the mobile gene (see comment 5 above)

For simplicity in our main model we assume a fixed cost (c) to resistance regardless of mobility. In response to the Reviewer's query we have now included an additional analysis in which we set a substantially smaller cost for effectively immobile resistance genes than more mobile ones (setting a cost of $c^{immobile} = 0.1*c$ for $\gamma = 10^{-6}$). As shown in our new figure below, this also does not qualitatively change our results:

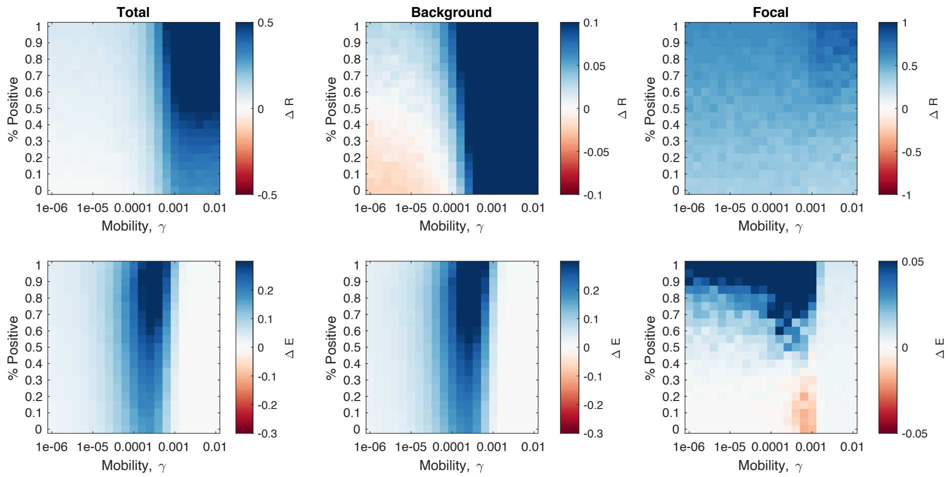

Figure S10. Allowing smaller costs to immobile genes do not qualitatively change our results. As immobile genes are often less costly than mobile ones, here we repeat the analysis in Fig 3, but allow a reduced cost for the least mobile resistance gene (setting $c^{immobile} = c*0.1$ for the case of $\gamma = 10^{-6}$). Throughout patch color represents mean ΔR or ΔE over 25 independent, 10-species communities, across a range of 21 Positivity and γ values. Other model parameters given in Table 1.

7. L 103: "equilibrate in the presence of a low level of the stressor,": What was the time period of this relative to the pulse and period after the pulse? In the simulations, the equilibration period went on till the 'background community' was 100% plasmid-containing. That seems rather extreme in a natural community with a low level of selection. Referring to comment 1, it is of course to be expected then that all the background species do not decline in abundance much after the pulse of stressor since they all acquired the resistance gene by then. In the experiment the equilibration period was 40 days (or 10 transfers), and the fraction of resistant cells was probably a lot lower than after that period in the simulations (not shown, see major comment 2 above).

The reviewer misunderstands the modelling set-up: As outlined in response to comment 2 - our simulations do not wait until the community is 100% plasmid positive. Indeed, as illustrated by Fig 2C, for the majority of parameter space without prior selection only a small fraction of initially susceptible cells have acquired the plasmid prior to the perturbation (this fraction is higher with prior selection, but remains small).

Instead, each community is exposed to the low-level stressor for a fixed period of 500 time units, creating a roughly equivalent ratio of low:high stressor exposure to that seen in our experiments (500:25 vs 40:4). Increasing the period of equilibrium would increase, although in most cases still not saturate, this fraction, but the core conclusions would remain the same.

We have now adjusted our text to make this point clearer:

> *"We calculate the stability of any given community by simulating its dynamics in response to a perturbation. Specifically, we* ***first solve the community dynamics for an initial "adjustment period" of t = 500 time units, allowing the community to reach an approximate steady state in terms of taxa abundances (importantly, while typically also effectively stable, resistance gene frequencies may not be at a true***

***equilibrium). We** then briefly perturb **the community** by setting the stressor level within the environment to D = 0.1 for t = 25 time units”*

Finally, as outlined above, the fraction of resistant cells in our experimental system was already shown for the no-prior-exposure case (Fig 4B) and as shown above, we have now added a new figure (Fig S6) showing equivalent results for the prior-exposure case.

8. In Figure 2D: I don't remember seeing an explanation for why there was no positive delta E when the gene was 'super mobile' (gamma -2)? Similarly, in Fig. 3 E-G. It seems that the transfer rate is already so high (see point 1: unnaturally high) that selection by a prior exposure to the stressor is not helpful?

As the reviewer has correctly determined, when plasmids are “super mobile” they spread widely within the community even in the absence of any prior exposure, thus there is no longer any benefit from prior exposure. We have added a line to explain this now:

*“As a consequence, prior exposure substantially increased the stability of background species (Fig 2D, Δ**E > 0). Notably, this effect was strongest for intermediate-mobility resistance genes, as highly mobile resistance genes spread within the population even without prior low-level stressor exposure, whereas low-mobility resistance genes remain at relatively low frequency in background taxa even with prior low-level exposure.”***

It is important to note that none of our major conclusions are based on this “super mobile” plasmid behavior. However, we believe it is important to show results that cover a full range of parameter space.

Minor comments:

1. L 22: I think this should read more like 'particularly for genes on mobile genetic elements with high transfer rates', as the genes don't really have a transfer rate.

We have adjusted this accordingly

2. L 22: "However,...": the 'mobility' aspects is not contradictory to the statement above. Why 'However'?

Here we aim to emphasize that while the impact on overall community stability is positive, the impact of resistance genes on individual taxa may in fact be negative.

3. L. 28: "counterintuitively,": Is it really counterintuitive? As a donor (focal species) shares its resistance genes it is giving possible competitors a benefit they didn't have before ('competitive release', as explained on L.225 and 267). It seems logical that some of these donors may 'suffer' from their 'altruistic donation' of their resistance gene.

We have removed the word “counterintuitively”.

4. L 103: "equilibrate in the presence of a low level of the stressor," What does that mean in

the model? That was not clear at this point. How are the growth rates affected by this low level of stressor? I first thought that you were allowing for resistance mutations to arise and be selected, but only later on it became clear that this low level selects for transconjugants in the background community that acquired the resistance gene.

The reviewer is correct in their understanding and we have adjusted our text to make this clearer:

> *we could simulate the scenario in which a given microbiome **first has a fixed period of time to adjust to a new environment, and** is then briefly exposed to an external stressor such as a heavy metal or an antibiotic perturbation (Fig 1B)...*
>
> *…First, we simulated microbiome dynamics when all taxa were susceptible to the stressor, and then again when a randomly chosen focal taxon carried a gene **encoding resistance to the stressor. Depending upon its mobility, this resistance gene could spread into susceptible cells during both the initial adjustment period, and during the perturbation window.***

5. Same problem on p. 31: what is the effect of prior exposure? It would be good to define that you are assessing the effect of prior selection, allowing a higher plasmid-bearing fraction.

Please see above, we have adjusted our text to clarify.

6. L. 134: "enabling it to spread into the background community even at lower rates of gene mobility": Here is an example of where it should be clarified that this is simply thanks to prior selection on the transconjugants formed in the period before the pulse.

We have adjusted this to now read:

> *"…enabling **mobile resistance genes to spread and reach low but non-zero frequencies in** background taxa even at lower rates of gene mobility"*

7. L. 223: again, here too, this doesn't seem counterintuitive to me, as the other species are now also receiving the resistance gene and thus more competitive; as the authors state "limiting the benefits of competitive release."

We have removed this "counter-intuitive"

8. L. 228, 236: Here again, this is all explained by the stability of the focal species as it is the only one that is resistant when the gene is not mobile. And (L 236): "reflecting that increases in stability were dominated by the behavior of the focal species": in other words: the one focal species survived the mercury pulse and the others didn't.... in spite of the resistance gene in that focal strain being mobile, but not mobile enough. See major comment 3. This is not surprising and can hardly be called community stability.

As outlined in comment 3, that different taxa are affected in radically different ways depending upon the ecological and evolutionary properties of the community is a key point that we wish to convey - and we believe this pattern is not necessarily to be expected. This is precisely why we analyze community stability averaging across the entire community but also across background and focal taxa separately. As discussed above, we have edited our text to make this point clearer.

9. L. 259: 'resistance gene' would be more clear than 'resistance'

We have adjusted the text accordingly

10. L. 264: "with prior exposure increasing the stability of the focal species if it was sensitive": How can prior exposure have any differential effect when there is no resistance gene? Is it purely due to different tolerance to Hg and depends on who is in the background community?

The reviewer is correct, this is likely driven by the sensitive focal taxon having already dropped somewhat in abundance due to the low-level mercury perturbation, such that the fold change in response to the mercury pulse is lower. We have adjusted our text to comment on this:

> *"And, notably, this decreased stability* ***of the focal taxon*** *was* ***not observed in communities where*** *the focal taxon* ***did not carry the resistance gene, likely due to mercury susceptible SBW25 having already been driven to low abundance by prior low-level exposure to mercury before the perturbation****."*

11. L.585, Figure 1 legend: the first 'competition' should red 'cooperation'

Thanks for catching this, we have adjusted accordingly

12. L. 594: is weak and low-level defined somewhere, and aren't they referring to the same? How much does it decrease the growth rates?

Weak and low-level refer to the same thing, thus we have adjusted our text to use low-level consistently throughout. As defined in the methods, low-level selection has 1/10th the impact on growth as high-level selection.

Reviewer #3
In this paper, mathematical modelling and a soil microcosm experiment are combined to study how ecological interactions and Horizontal Gene Transfer (HGT) of resistance genes shape the response of different microbes to perturbation/stress. After carefully reading this paper, I am left with mixed feelings. On the one hand, the paper shines in describing the intricacies of the interplay between HGT and ecological interactions, and in showing how these factors affect the prevalence of microbes originally harbouring a resistance gene ("focal species") and other microbes ("background species"). These are novel and important results, and I find the combination of model predictions and experiments very strong. On the other hand, I have major concerns about the current focus of the paper (especially with regard to the broad claims made about "microbiome stability", which I find unjustified). Also, several results warrant further explanation and/or critical re-examination. I therefore recommend major revisions.

We are very happy that the reviewer enjoyed our paper so much and, as outlined below, believe we can readily address each of their concerns

Major comments – necessary to address

(1) Microbiome stability

The narrative is currently structured around the concept of “microbiome stability”, where stability is defined as the (lack of) change in abundance of certain microbes (focal, background, or all) in response to an external perturbation. Hence, “stability” means “stability against the external perturbation”, or in other words “lack of effect of the external stressor on bacterial abundance”. This is highly similar to resistance, since the resistance gene reduces the negative effect of the stressor on the bacteria. Hence, resistant bacteria should show a smaller drop in abundance when under this stress, and we should expect that a higher prevalence of the resistance gene leads to a higher stability. Especially in the case without ecological interactions, this similarity between resistance and stability easily leads to circular arguments.

While interlinked, resistance and stability are fundamentally different concepts. Resistance in the sense referred to by the Reviewer is a property of a single cell in response to a specific stressor. Stability is a property - typically of an entire community - that reflects not only the individual direct impact of a stressor on each cell, but also the net indirect impact on abundances driven by changes in individual community members and subsequent feedback loops.

Crucially, these two measures do not go hand in hand: For example, in interacting communities (as most microbiomes are likely to be) we see that the stability of susceptible community members can increase without them acquiring resistance (i.e. in the case of cooperative communities, upper left of Fig. 3C), while resistant species may decrease in stability even when their resistance remains the same (i.e. in the case of prior exposure in some competitive communities, lower part of Fig. 3G).

We have adjusted our text to make this distinction more clearly:

> *“Although simple, these gLV models have been shown to well capture and predict the dynamics of both host-associated and environmental microbial communities[14,18–21].* ***However, previous work on the stability of these communities in the face of perturbations has assumed all taxa within any given microbiome are equally impacted by environmental perturbations such as antibiotics[13,14]. That is, microbiome stability has typically been assessed by examining how communities respond to uniform, instantaneous changes in each constituent taxa’s abundance. As our goal is to explore dynamic variability in the impact of stressors on individual microbial taxa owing to resistance genes, we now*** *extended this basic model to explicitly incorporate a stressor that inhibits (or kills) susceptible cells and a potentially mobile resistance gene that protects cells encoding it, but at the cost of a reduced intrinsic growth rate.* ***This adjusted model allowed us to assess microbiome stability in the face of perturbations when the susceptibility of individuals to those perturbations varies between taxa and dynamically over time.***
>
> *…****More specifically, we focused primarily on one key measure of stability: the average decrease in taxa abundances following a perturbation, often termed community Robustness (Fig 1B). This measure allowed us to quantify the immediate response of the community to a perturbation. However, for completeness, we also quantified two further metrics of stability – the change in community composition induced by the perturbation (measured as Bray-Curtis dissimilarity), and the time taken for the community to return to its original state following the perturbation. Each of these alternative stability metrics produced qualitatively similar results (see Supplementary Information and Figures S1 and S2).”***

Under these conditions, the carrying capacity of a species linearly depends on its growth rate r_i and since the perturbation is modelled as a reduction in growth rate (and this reduction is less severe for resistant bacteria) resistant bacteria will necessarily show a smaller drop in abundance due to the perturbation (see attached appendix – part A). Line 121 ("Introducing any resistance gene increased overall microbiome stability") is hence not a general result, but a direct consequence of the way stability is defined and the way the perturbation is modelled. This should be clear from the text. Overall, the authors can do a much better job at explaining the similarity between (their definition of) stability and resistance throughout the manuscript. More care should be taken to separate results from model assumptions.

Please see above for our discussion of why individual resistance and community stability are distinct concepts that capture different dynamics, alongside adjustments to our text to make this distinction clearer.

Because of the narrow way the authors define stability (in terms of resistance against a specific perturbation), I find the broad claim that "HGT increases microbiome stability" unjustified. Using the authors' definition of stability, this statement could be rephrased to "HGT facilitates resistance" (through increasing the spread of resistance genes). This is neither a novel nor a very surprising result. In my opinion, the novelty of this work lies in studying the interaction between ecological interactions and HGT of resistance genes, which yields exciting and interesting predictions on the dynamics of resistance-harbouring and (initially) sensitive strains in communities with different types of ecological interactions. The paper would be much stronger if the focus was shifted, and the title changed.

Again, we really must strongly emphasize that individual resistance and overall community stability are fundamentally different, and that we have now adjusted our text to clarify this point.

That said, we do agree with the reviewer that the exciting and interesting predictions regarding the interaction between ecological interactions and HGT did not come across strongly enough in our original manuscript. As such, we have changed our title to:

> ***"Horizontal gene transfer and ecological interactions jointly control microbiome stability"***

Alongside editing our text at various points throughout to emphasize the more interesting results. For example:

> *"However, the impact of resistance genes upon the stability of individual taxa varies **dramatically** depending upon **the identity of individual taxa**, the mobility of the resistance gene and the network of ecological interactions within the community."*

> ***"Remarkably however, w****hile mobile resistance genes **increased** overall microbiome stability, examining focal and background taxa separately revealed **radically** different **impacts of the resistance gene on individual taxa. That is,** background and focal species **showed** markedly different responses to resistance genes depending upon the precise manner in which taxa were interacting and the mobility of the resistance gene.…*

> ***"…This stark difference in dynamics between the community as a whole and that of background taxa reveals the critical importance of looking beyond average***

community properties when studying microbiome stability. Meanwhile, the dramatic differences between communities with differing interaction types underlines the vital role of ecological interactions in shaping overall microbiome dynamics."

The definition of stability used here is not the only one possible. For example, in theoretical ecology stability of a community usually refers to the linear stability of the equilibrium (this is e.g. the case in references 13 and 14). It is not obvious that results obtained for one definition of stability would transfer to other definitions. In the supplement, the authors address this issue by repeating their analysis with two other diversity measures: Bray-Curtis dissimilarity and recovery time. These results are however not discussed in the main text, which I find odd.

Given each of our stability metrics reached the same core conclusions we did not see a need to discuss these in depth in the main text. We have however adjusted our main text to make clearer that the stability metric we are calculating is often also referred to as community Robustness (see new text above).

As discussed in more detail below, there are numerous possible metrics of stability - we have chosen three that best match the biological scenario that we are interested in, namely how communities respond when taxa are not equally affected by a given perturbation.

Furthermore, there are two major issues with these additional results:

(I) The definition of recovery time used in the analysis seems to be incorrect, and this mistake biases the results to resemble the results obtained in the main text. Recovery time is usually calculated as the average time taken to return to equilibrium given a fixed perturbation in the bacterial abundance. Mathematically it is defined as the derivative of dx/dt towards x around the equilibrium (see e.g. Box 2 of Scheffer et al., Nature, 2009). From lines 10-11 of the Supplementary Information, it appears that the recovery time in this instance is calculated based on how long it takes for a community after perturbation to recover to the pre-perturbation equilibrium. If the community is further away from its pre-perturbation equilibrium (because the stability as defined in the main text is lower), it will evidently take more time to get back to the equilibrium. This does however not imply that its recovery time is also larger.

As the reviewer mentions in their earlier comment and alludes to above, a commonly used method of quantifying stability is assessing how communities respond to a small, fixed perturbation in species abundances about an equilibrium. Often this is determined analytically by examining the systems eigenvalues (linear asymptotic stability, LAS), with more negative eigenvalues implying that the system has a faster "Recovery *Rate*".

However, as detailed in our introduction and noted by the Reviewer themselves, LAS and its numerical equivalents each assume that all species are equally affected by a given stressor (i.e. fixed perturbations in abundances). The precise goal of this work is to examine what happens when species are NOT evenly affected by a stressor, and moreover what happens when this susceptibility changes dynamically over time. That communities which are more susceptible, thus more perturbed, therefore take longer to return to their original state is precisely the sort of dynamics that we are interested in.

Regarding the use of the term "Recovery *Time*" specifically, this term is used variably across the literature, and while it does map to LAS ($-1/\max(\mathrm{Re}(\lambda))$, sometimes termed Resilience, though this too is variably defined), it is more generally used simply to describe the time for a community to recover from a perturbation, without strict requirements on perturbation size, thus is perfectly applicable to our scenario.

Please see our response to comment (I) for the new text we have added to clarify this.

(II) It is not clear if the calculation of Bray-Curtis dissimilarity was based on relative or absolute abundances. If the Bray-Curtis dissimilarity was calculated based on absolute abundances, there is a positive relation between the stability as defined in the main text and the Bray-Curtis dissimilarity (see attached appendix – part B.). Hence, similar results should be expected. This should be mentioned.

As per its definition, our Bray-Curtis dissimilarity is based on absolute abundance counts. However, while Robustness and BCD are certainly related, there is not a one-to-one mapping between the two as stated by the reviewer.

Trivially, consider two two-species scenarios:

1) Abundance before = [3, 3], Abundance after = [2, 2], Robustness = -1.8, BCD = 0.2
2) Abundance before = [3, 3], Abundance after = [1, 3], Robustness = -2.4, BCD = 0.2

As you can see, we see identical Bray Curtis Dissimilarity between these two scenarios, yet one is more Robust than the other. We think the discrepancy between our example and the Reviewer's comment might stem from a typo in the Reviewer's appendix accidentally setting sum(X_i/Y_i) to sum(X_i)/sum(Y_i).

We focus on Robustness in our main text as we believe it is the more intuitive measure, and can be calculated for the community as a whole and individual taxa in isolation. However, we also included the Bray-Curtis analysis as this metric is so commonly used in experimental microbiome work.

(2) Equilibration and maintenance of resistance gene

If I understood correctly, all simulations were first run to reach equilibrium without the external stressor (or with low stress), after which high stress was applied for the duration of the perturbation. It is however not clear to me how the resistance gene was dealt with during the equilibration period, and how equilibrium was then defined. Some explanations given in the main text (e.g. lines 128-136) suggest that the resistance gene dynamics were included in the equilibration period. This however raises two questions:

We apologize for some confusion here, as the reviewer correctly notes at the end of this section, we used equilibration period to refer to a fixed period of time during which community abundances are solved for prior to any perturbation. To allow a fair comparison we use the same period of time for each community with or without a resistance gene, with or without prior selection. This means that in some cases - particularly in the presence of the low-level stressor - species abundances may be at an equilibrium but resistance genes may not. We have adjusted our text

in several areas to clarify this point for the reader, and for simplicity have entirely removed the term "equilibration period":

> *"More specifically, we could simulate the scenario in which a* ***given*** *microbiome* ***first has a fixed period of time to adjust to a new environment, and*** *is then briefly exposed to an external stressor such as a heavy metal or an antibiotic perturbation (Fig 1B)."*
>
> *"First, we simulated microbiome dynamics when all species were susceptible to the stressor, and then again when a randomly chosen focal species carried a gene encoding resistance to the stressor.* ***Depending upon its mobility, this resistance gene could spread into susceptible cells during both the initial adjustment period, and during the perturbation window****."*
>
> *"We calculate the stability of any given community by simulating its dynamics in response to a perturbation. Specifically, we* ***first solve the community dynamics for an initial "adjustment period" of t = 500 time units, allowing the community to reach an approximate steady state in terms of taxa abundances (importantly, while typically also effectively stable, resistance gene frequencies may not be at a true equilibrium). We*** *then briefly perturb* ***the community*** *by setting the stressor level within the environment to D = 0.1 for t = 25 time units"*

Importantly however, while altering the length of the adjustment period will incrementally alter the magnitude of the effects seen, unless this period is extremely long our results remain qualitatively identical (more on this below).

(I) Without pre-conditioning (low level stress during equilibration), how was the resistance gene maintained in the population? A back-of-the-envelope calculation shows that for the parameter values of Table 1 the resistance should be lost completely from the population (see appendix – part C; derivation similar to van Dijk et al., eLife, 2021). Why does this not happen?

Please see above re definition of equilibration. Further, while the reviewer correctly identifies that in a single species system a plasmid will eventually be lost unless the rate of transfer exceeds the cost, in a multispecies system the transfer of plasmids from the focal species into the background community is typically sufficient to maintain a low level of plasmid within the background community at equilibrium. While we cannot solve for this analytically in an interacting, multispecies system, we have previously shown this dynamic analytically in a simpler "source-sink" model of plasmid dynamics (Hall et al PNAS 2016). Perhaps more importantly, this is also evident in our experimental data (Figs 4B, S6).

Of course, the Reviewer is entirely right that the plasmid may still be lost in some cases - however, this requires high rates of plasmid loss via segregation and/or long periods of adjustment, and we believe an in-depth exploration of this is beyond the scope of this study.

(II) Why is there a difference between focal and background species in resistance carriage after equilibration? Since HGT-rates are similar between all species, given enough time I would expect the prevalence of the resistance gene to be similar in the focal species and the background species.

Again please see above re our definition of the equilibration period (now termed "adjustment period" to avoid confusion).

Moreover, as evident in Fig 2C, at high levels of gene mobility (a good proxy for long adjustment times) the prevalence of the resistance gene is indeed similar between the focal and background species.

These two points suggest to me that while the species abundances might be in equilibrium after the equilibration period, the dynamics at the gene level are not. If this is true, the definition of "equilibrium" should be adjusted (e.g. in lines 103 and 365).

Furthermore, this would also suggest that a longer equilibration period could affect the results (giving the gene more time to spread to the background species before the stress is applied), which should be discussed. Addressing this is not just a theoretical exercise, but also bears real-world significance: if exposure to the stressor is rare, one would expect long periods between exposures, and resistance genes are at high risk to be lost. If however exposure is relatively common, one should expect genes to be maintained at higher costs and/or lower HGT rates. These considerations are furthermore important in the interpretation of the results on HGT in highly competitive communities: the explanations relying on differences in competitive release between focal and background species (lines 172-179 and 187-190) rely on differences in the prevalence of resistance in the focal and background species prior to the perturbation. In other words, these explanations rely on the system not being in equilibrium at the gene level.

As outlined above, we have now adjusted our text to reflect this important distinction between species and gene level equilibria. It is important to note, however, that our results do not rely on the system being in a non-equilibrium state - as we have already previously shown, source sink dynamics between species are sufficient to maintain costly plasmids at equilibrium (Hall et al 2016).

That said, the reviewer is entirely right that in cases of long equilibration periods combined with either identical, high conjugation rates between species, or high rates of plasmid loss the prevalence of resistance will be identical between focal and background strains. As such, we have adjusted our text to highlight this fact (see above and below). However, given these are likely to be rare scenarios an in-depth exploration of this is beyond the scope of the paper.

> *"However, in certain microbiomes spread of the resistance gene may be costly for the original host – reducing its advantage over otherwise susceptible competitors[33].* ***Which scenario plays out in any given system will depend upon the interplay between the ecological and evolutionary properties of the underlying microbiome, and the nature of the environment these communities inhabit. Our study is far from exhaustive, and exploring how these eco-evolutionary dynamics play out between different scenarios such as rare versus common, or static versus fluctuating perturbations – each of which have been shown to play important roles in shaping HGT in single species populations[34] – offer exciting future avenues for research."***

(3) Tuning of growth rates and relationship between rates & duration of perturbation

In the Material & Methods (line 360), it is described that growth rate values r_i are tuned such that all species approximately have abundance 1 at equilibrium (in the absence of resistance genes). This assumption might have important consequences.

First of all, the growth rate r for species that are preconditioned with low-level stressor is higher than the growth rate for species that are equilibrated without the stressor. This is easily seen in the absence of ecological interactions: the equilibrium abundance of species i is equal to (r_i –

betaS*D) / s_i. Including a value D>0 during equilibration will require r_i to be increased to still reach abundance 1. It is not directly clear to me if, and if so how, this affects the results. My expectation is that it may reduce the effect of the later pulse of high stressor levels, because the relative reduction in growth rate caused by the stressor is lower. If this is true, this might explain the surprising difference in stability between -Exposure and +Exposure for -Resistance gene in figure 1D. It would be worthwhile to explore this effect.

There has been some confusion here – we keep the same underlying community properties (e.g. r_i / s_i) in the presence or absence of the stressor. We do not adjust r_i when the stressor is present as the reviewer suggests, rightly, that this would be an inappropriate choice. We have altered our text to clarify:

> *"**in line with previous work,** we set the intrinsic growth rates r_i such that **in the absence of any stressor** the community has a linearly asymptotically stable equilibrium at…"*

Similar considerations hold for the difference between competitive and cooperative communities. If tuning is done in a similar way (which I assume it is), growth rates will generally be larger in cooperative communities than in competitive communities (see appendix – part D). Again, it is not directly clear to me if, and if so how, this will affect the results. However, given that the perturbation is applied for a fixed amount of time (25 time units), higher growth rates might lead to either faster decline of the community than would be the case if the growth rates were similar, or to faster adjustment of the community to an equilibrium with a higher prevalence of resistance. Either way, it would be good to verify that this modelling assumption does not majorly affect the results.

The reviewer's calculations don't quite match up to ours (we solve for the intrinsic growth rates by solving r= -M*X, with no requirement on r_i=r_j). However, they are entirely right to note that differences in growth strategies between cooperative and competitive communities are an important aspect of understanding microbiome dynamics. Specifically, in solving r= -M*X we enforce a trade-off whereby species maintain equivalent abundances either through investment in intrinsic growth (high r_i) or through investment in cooperative growth with others (high a_ij). Both species types are equally directly affected by the perturbation, however, cooperative species also suffer from the loss of their cooperative partners, creating negative feedback loops that further enhances the knock on effects of the perturbation. This nuanced trade-off sits at the heart of much of theoretical ecology, but is very much underappreciated so we are happy to see the reviewer discuss it (see the supplement of Coyte et al 2015 Science for more detail)

The alternative strategy would be to fix r_i for each taxon and remove any trade-off between growth strategies. Importantly, in this case our key findings would remain (stabilizing effect of plasmid spread overall, destabilizing effect of immobile resistance within competitive communities etc). However, this would also enforce entirely costless cooperation, and lead to Bob May's unrealistic "orgy of mutual benefaction". To analyze this model correctly would therefore in turn require vast scans of parameter space to find realistic, feasible cooperative communities. While it might be interesting to explore whether we can find scenarios here that go against our core conclusions, given they will be rare and the unbiological nature of this alternative approach, we think this is beyond the scope of this paper.

As a nod towards this interesting discussion, however, we have edited our text to make this trade-off even more clear:

"Importantly, setting the intrinsic growth in this manner introduces a trade-off in growth strategies, with taxa investing either in their own intrinsic growth rate, or growth via cooperation with others, reducing unrealistic explosive behavior in highly cooperative communities."

More in general, I expect the size of the effect of HGT on DeltaR and DeltaE to depend on the duration of the perturbation. Namely, as long as the resistance gene is present and HGT occurs at some level prior to the perturbation, resistance will be selected for and spread. If the perturbation extends for a really long time, all cells will be resistant at some point and DeltaR will no longer depend on gamma. The speed at which the gene can jump from focal species to background species, and within species, does depend on the HGT rate. There is likely an interplay between HGT, the perturbation duration, and the size of the effect found.

The reviewer is right that in the case of extremely long perturbations the dependence on the magnitude of gamma will be removed. However, it's important to note this will only apply to cases where the susceptible cells have been entirely wiped out, and the core difference between plasmid-encoded versus chromosomal genes will remain.

Major comments – recommendations

(4) Mechanisms through which HGT facilitates resistance/stability

As shown in figure 2B and C, the increase in stability (positive DeltaR) corresponds to an increase in the prevalence of resistance. In my view, HGT can facilitate resistance (= stability) in two ways:

(i) By increasing the prevalence of the resistance gene prior to the perturbation, such that the population is more robust against the stressor to begin with; and

(ii) By increasing the spread of resistance during the perturbation period, speeding up the generation of resistant cells, that are subsequently favoured by selection.

I think that disentangling these two processes will allow for a deeper understanding of the nature of the effect of HGT on resistance. I therefore strongly recommend plotting the proportion of resistant cells in focal, background and total populations both prior to and after the perturbation. These plots should be relatively easy to make, but I expect they will provide more insight into what HGT actually does in the system, as well as showing the effects of pre-conditioning. It would also provide a measure / sanity-check on how the equilibration time affects prevalence of resistance (see point 2).

The reviewer is correct that both mechanisms are at play here, and the majority of their suggested plots are already included in our original manuscript (e.g. see Fig 2C for plots of resistance prior to the perturbation in the focal, background, and overall community with and without pre-conditioning within the model, and Figs 4B, S6 for resistance before and after the perturbation, with and without preconditioning within the experimental system). We agree that further investigation of this point would be interesting, but feel this is beyond the scope of this manuscript.

(5) Embedding in existing literature

The discussion would benefit from a stronger embedding in existing literature. In particular, I recommend linking to results on the role of ecological interactions in the response of bacteria to stresses such as antibiotics (see e.g. de Vos et al, PNAS, 2017) and to results from models of HGT in microbial communities (see e.g. Hernandez-Bertran et al., Front Microbiol, 2021 for a recent review).

We have added a statement on the role of ecological interactions in shaping resistance without requiring HGT (citing de Vos 2017, new reference 36) and discussed some of the outcomes of previous theoretical work (citing Hernandez-Bertran 2021, new reference 34):

> *"Similarly, our model does not explicitly include de novo mutations, phenotypic* ***plasticity, or social interactions***[36] *that may modulate how individual taxa respond to stressors without requiring the acquisition of resistance genes from other community members…*
>
> ***"Our study is far from exhaustive, and exploring how these eco-evolutionary dynamics play out between different scenarios such as rare versus common, or static versus fluctuating perturbations – each of which have been shown to play important roles in shaping HGT in single species populations***[34] ***– offer exciting future avenues for research"***
>
> ***"A large body of theoretical and experimental work has explored how HGT modulates the dynamics of single-species populations***[34,38]***, and the challenge now is to understand how such species-level effects scale-up to control the dynamics of complex microbiomes."***

Minor comments

(6) The stability of the background or full community is defined as the mean stability of all members of that group. It is unclear to me what happens if some species actually increase in density due to the perturbation, rather than decrease. Can this occur (for instance in communities with strong interactions)? And how is this then incorporated in the calculation of mean stability?

The reviewer is right that in strongly interacting competitive communities occasionally some species will slightly increase in abundance during the perturbation. As a "negative decrease" is a bit misleading, we treat cases where abundances increase as a reduction of zero. However, using the raw change in abundance instead (ie including rare instances of increases) does not qualitatively change our results. We have edited our text to make this clearer:

> *"We define the stability of each individual species i based on their fold change in response to the perturbation, Stability=log10(Aa/Ab),* ***such that more negative values indicate less stable species (for simplicity, we set values of Stability greater than zero to zero, although dropping this step does not qualitative change our results)****."*

(7) Throughout the text and figure legends, references are made to Table 1 (parameter values). Since the interpretation of some results depends on the parameter conditions (see e.g. some of my comments above), I find it highly unfortunate that this table is currently placed in the Supplementary Information. Please consider moving it to the Materials & Methods.

We have now included Table 1 in the main text.

(8) In lines 310-316, it is stated that the model does not include differences in HGT-rate between closely related and more distantly related species, although such differences have been observed. This could however easily be included in the model by adjusting the gamma-values based on phylogenetic distance. I do not think it isnecessary to conduct such simulations for this manuscript (although it might allow for an even better, more detailed match between a set of simulations and the soil microcosm experiment), but why is this “easy fix” not mentioned?

We had originally considered including some form of phylogeny (and phylogeny dependent conjugation rates) in our original model, but ultimately elected not to as we felt that imposing an explicit phylogeny upon this type of model is somewhat arbitrary. However, instead we did explore the idea of differences in conjugation rates depending upon how taxa interact with one another – capturing differences in conjugation depending on taxonomy without requiring additional assumptions about phylogeny.

More specifically, while our main model assumes that HGT can occur between any given pair of species, we also modelled three further scenarios: HGT only between interacting species, HGT only between competing species, and HGT only between cooperating species.

As shown in the figures below, these scenarios subtly shift the impact of HGT on microbiome stability, however, our primary conclusions remain the same. We have now included each of these additional figures in our Supplementary Information, and adjusted our main text to highlight their results:

> “…with more distantly related members of the community such as *B. megaterium* apparently unable to gain the resistance genes, suggesting they were unable to acquire or maintain either plasmid. **Embedding simple constraints on conjugation within our model (SI Fig 7-9) reveals that differences in transfer probabilities between taxa can subtly modulate the impact of mobile resistance genes upon community properties.”**

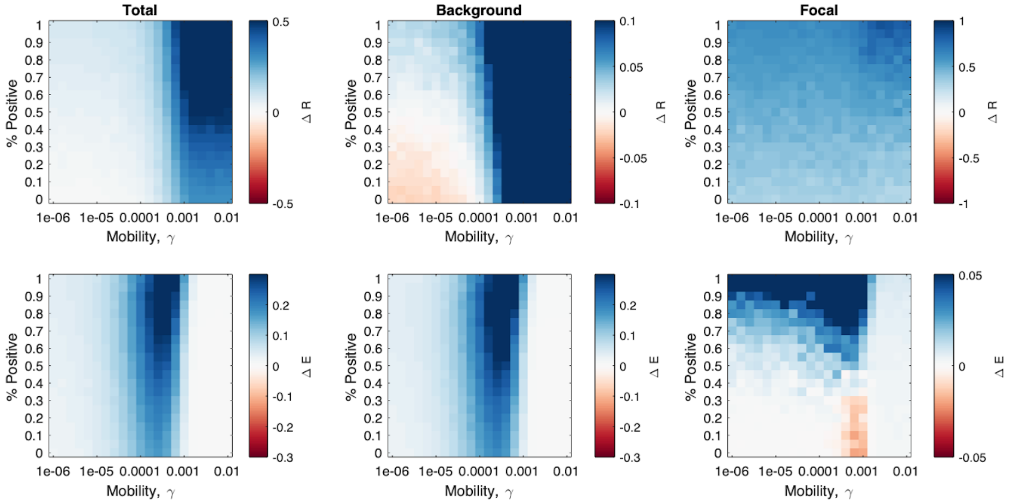

Fig S7. Modelling HGT only between interacting taxa. Recapitulating Fig 3 analysis when resistance genes only transfer between taxa that are interacting ecologically ($\gamma_{ij} > 0$ if $|a_{ij}|$ or $|a_{ji}|$ >

0). Patch color represents mean ΔR or ΔE over 25 independent, 10-taxa communities, across a range of 21 Positivity and γ values. Other model parameters given in Table 1.

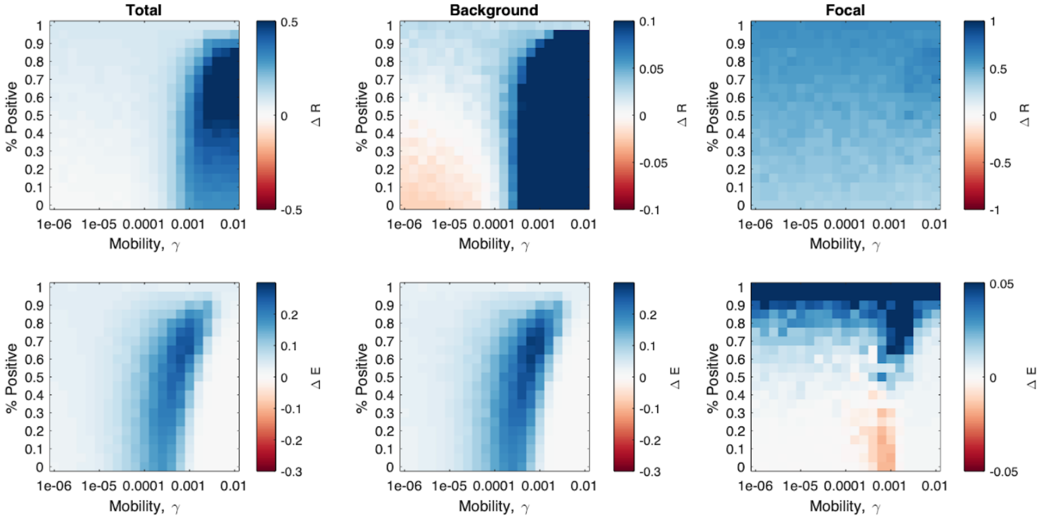

Fig S8. Modelling HGT only between negatively interacting taxa. Recapitulating Fig 3 analysis when resistance genes only transfer between taxa that are interacting negatively (γ_{ij} >0 if a_{ij} or a_{ji} < 0). Patch color represents mean ΔR or ΔE over 25 independent, 10-taxa communities, across a range of 21 Positivity and γ values. Other model parameters given in Table 1.

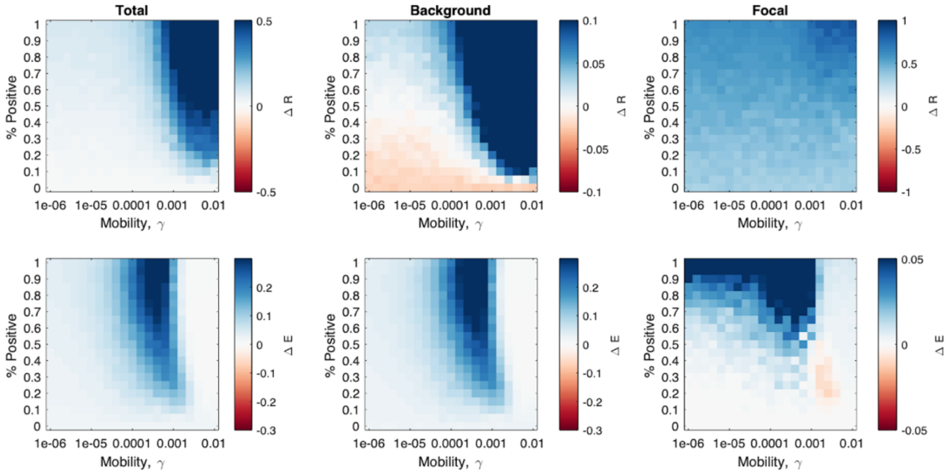

Fig S9. Modelling HGT only between positively interacting taxa. Recapitulating Fig 3 analysis when resistance genes only transfer between species that are interacting positively (γ_{ij} >0 if a_{ij} or a_{ji} > 0). Patch color represents mean ΔR or ΔE over 25 independent, 10-taxa communities, across a range of 21 Positivity and γ values. Other model parameters given in Table 1.

Details

Lines 84-86: Three different terms are used to describe microbe type (“species”, “kin”, “taxon”). It would be more consistent to pick one.

We have adjusted our text to use “taxa” / “taxon” throughout

Line 346: Indexing of j = 2:N assumes that i = 1 (while this is not defined).
Thanks for catching this

Line 358: Superfluous “is” between “a_ij” and “from”.
Thanks for catching this

Line 359: Sigma = 0.05 is mentioned here, while according to Table 1 sigma = 0.015. Which is correct?
Thanks for catching this, we have corrected this to read 0.015

Line 359: r_ij should probably be r_i?
Thanks for catching this

Line 585 (Fig 1): “competition or competition” à “competition or cooperation”.
Thanks for catching this
