# Supplementary material for: Horizontal gene transfer and ecological interactions jointly control microbiome stability
Source: PLoS Biol. 2022 Nov 9;20(11):e3001847. doi: 10.1371/journal.pbio.3001847 (PMC9678337; doi:10.1371/journal.pbio.3001847)
Supplement: Attachment — Submitted filename: reviewer_response_august23.pdf [file pbio.3001847.s004.pdf]

We thank all the reviewers and editors for their useful comments throughout the review process, we believe our manuscript is now far stronger following their feedback. As suggested and outlined in more detail below, we have addressed each of Reviewer 1's final queries, and included a short paragraph discussing our results in the context of human health.

**Reviewer #1:** The authors thoroughly responded to each comment and adjusted the manuscript accordingly. I apologize for some of my earlier misunderstandings, but I must say that the manuscript is more clear now with the additional clarifications.

I have just a few last minor comments:

1. L. 413: "...16S rDNA gene amplicon sequencing": This should really read 'by amplicon sequencing of the 16S rRNA gene'. (There is no ribosomal DNA).

Thanks for catching this, we have adjusted our text as suggested.

2. L. 418: "After adjusting to microcosm conditions, each microbiome contained between three and five taxa at appreciable abundances". Does this mean that more than 90 of the added strains were no longer detectable? That is worth pointing out I think.

No, it does not mean that >90 taxa were lost. There were 14 OTUs present in the original microbiome community (i.e., among the 96 random colonies there were multiple representatives of the same OTU). This was originally specified in the Methods at line 541, but we agree it is worth also mentioning this in the main Results section to improve clarity. As such we have added the following:

> "Specifically, we generated model microbiomes by inoculating sterile potting soil microcosms with 96 soil bacterial isolates (**representing 14 unique OTUs**, our background community)"
>
> "After adjusting, each microbiome contained between three and five detectable taxa, namely: OTU_107 (the focal taxon, P. fluorescens SBW25), OTU_3 (Pseudomonas sp.), OTU_14 (Pseudomonas umsongensis), OTU_9 (Bacillus megaterium), and OTU_19 (Bacillus simplex) **out of a total 14 OTUs present in the starting community**"

It would also be good to quantify 'appreciable abundances": what is the expected detection limit of the amplicon sequencing?

To clarify we have replaced "at appreciable abundances" with "detectable" (meaning the QIIME analysis returned a count > 0 for that OTU).

3. L. 451. Descriptions of Fig. 4C, D: I still think that there are a few experimental observations that are not entirely expected. The paper would benefit from pointing those out right away, in addition to pointing out the observations that are in line with the expectations. I think I see two or three:
a) "We also observed a positive impact of gene mobility on overall microbiome stability, however, this effect was small (estimated effect of mobility on stability, given resistance = 0.0065 [0.0017 - 0.011, 95% CI], BLM1), AND DID NOT CHANGE SIGNIFICANTLY WITH PRIOR LOW-LEVEL EXPOSURE TO MERCURY": If I understood the model output well, the latter points were not entirely expected based on the model: gene mobility had a large effect on total stability and prior exposure was supposed to select for horizontally

transferred resistance genes and thus have a larger positive effect on stability. Is it because overall stability is driven by that of the focal species and 'hides' the subtle changes in stability of the background?

The reviewer is correct – we believe that the non-significant effect of prior exposure in the total community is due to the dynamics being driven by the focal taxon. We have adjusted our text accordingly:

> *"We also observed a positive impact of gene mobility on overall microbiome stability, however, this effect was small (…), and notably did not change significantly with prior exposure (…)* ***suggesting that the large increase in total community stability conferred by the introduction of a resistance gene into the focal taxon masks the subtler changes in stability conferred by the transfer of resistance genes into the background community****"*

b) Fig 4D, E: the fact that the background community benefited from prior exposure (by quite a lot: orange dot vs blue dot) when the resistance gene was chromosomal (Fig 4D), and that the sensitive focal strain benefited from prior exposure (by quite a lot) needs some explanation. The authors bring some of this up in the Conclusions but it is puzzling the reader right here (around L. 497) when looking at Fig 4.

The reviewer makes a fair point here, we have now edited our discussion of the sensitive focal strain to make this clearer to the reader, and have added a short paragraph to our results section discussing the behavior of the background community in the chromosomal treatment:

> *"… This suggests that, as predicted by our models, the increased stability of the background taxa might indeed be reducing the competitive release experienced by the* ***resistant*** *focal taxon during perturbations, and thus decreasing the stability of the* ***resistant*** *focal taxon following prior exposure (cf. red region in Fig 3G).* ***In contrast, the finding of prior exposure increasing sensitive focal taxon stability was likely due to the sensitive focal taxon already having been driven to low abundance by prior exposure, thus weakening the subsequent impact of the perturbation."***
>
> *"****We did also observe some behavior not predicted by our model. In particular, in contrast to our modelling predictions, we found that prior exposure to mercury increased the stability of the background community in the presence of chromosomally-encoded resistance genes (Fig 4D). This suggests mechanisms other than plasmid transfer, such as de novo mutation, may also be playing an important role in microbiome stability. Nonetheless****, collectively,* ***our*** *experimental findings support* ***our*** *key theoretical predictions relating to the effects of resistance genes and their mobility upon the stability of competition dominated microbiomes."*

4. L. 778: "These were propagated by serial transfer of 1% of the community every 4 days into fresh soil microcosms for twelve transfers. It would help to understand that (i) this was sterilized soil, (ii) was it actually potting soil, or a slurry? (iii) When 'transferring 1%': is it 1% of the mass of the soil, or volume of soil or slurry?

The potting soil is sterilized; this was originally mentioned at line 503 but is now mentioned again at line 519. The medium is a potting soil not a slurry, now specified at line 512. We transferred 1% of a soil wash, now explained in greater detail at lines 519-523 and supported by a reference to published work that established the serial transfer protocol.

**Reviewer #2:** This manuscript is important to academic microbial ecology and, at the same time, directly relevant to human health. As the authors note and the editors agree, we know relatively little about the mechanisms determining the structure of microbial communities (microbiomes), their strain and species composition, the relative densities of bacteria in these communities, and the factors limiting their total densities and maintaining the stability of these communities. This study focuses primarily on the mechanisms responsible for the stable maintenance of these communities when they are confronted with stresses that kill and prevent the replication of the bacterias. From a human health perspective, the response to stress, like antibiotics, is particularly critical. Disruption of the gut microbiome stability leads to antibiotic-associated pseudomembranous colitis, which can have serious health and even lethal consequences.

The perspective presented in this report, that genes that code for resistance to the stresses and especially those that are horizontally transmitted by conjugative plasmids and temperate bacteriophage, is, to my knowledge, novel. Their model seems to provide a theoretical basis for this proposition, and their soil microbiome experiments support it. I also appreciate the combination of theory and experiments.

I support and recommend publishing this report in PLoS Biology, but only if the revised version of this report meet the following criteria:

I agree with almost all the extensive comments of the three editors that reviewed this report. For this paper to be acceptable for publication, these editors should tell PLoS Biology that they agree with the author's response to their reviews and the modification made in the manuscript to address their concerns.
pt.All three editors raised concerns about the criteria the authors use for the stability of the microbiome. I have concerns about that as well. I will be satisfied if the "editors" agree with how this issue is addressed in the current incarnation of this report.

In revising this manuscript, the authors should.

Consider the implications of this study to our understanding of the mechanisms responsible for maintaining the stability of the human gut microbiome.
Consider why stress-mediated disturbances of the human gut microbiome can manifest as a disease.
Briefly discusses that the gut microbiome disturbances resulting in pseudomembranous colitis can be successfully treated with fecal microbiome transplants.
Discuss the implications of horizontal transfer of antibiotic resistance genes, which this study considers to be responsible for maintaining the stability of the genome to the generation and dissemination of antibiotic-resistant pathogens.
Comment on the hypothesis that bacteriophages play a prominent role in determining the distribution and abundance of different species and strains of bacteria in the human microbiome.

We are very glad the reviewer enjoyed our manuscript and supports its publication. Following their suggestion, we have now included an additional final paragraph considering the implications of our study for human health, particularly in relation to attempts at microbiome engineering (eg via FMT):

> ***"As our knowledge of the ubiquity of microbial communities has increased, so too has our desire to manipulate these microbiomes for our own benefit – using microbiome transplants or individual "probiotic" bacteria to supplant pathogens, or simply to increase overall microbiome stability. Before we can engineer our microbiomes in a targeted manner, however, we need***

*means of systematically understanding and predicting how these communities change over time. While some theoretical work has explored the impact of ecological processes in determining microbiome dynamics, until now the impact of evolutionary processes such as horizontal gene transfer upon microbiome dynamics have been largely ignored. Our study shows that evolutionary changes can have profound and sometimes surprising impacts upon the ecological dynamics of microbial communities, and that accounting for both ecological and evolutionary forces will be critical if we wish to fully understand and ultimately manipulate microbiome dynamics."*
